# Uncovering the Mechanism of Action of Antiprotozoal Agents: A Survey on Photoaffinity Labeling Strategy

**DOI:** 10.3390/ph18010028

**Published:** 2024-12-28

**Authors:** Alessandro Giraudo, Cristiano Bolchi, Marco Pallavicini, Roberto Di Santo, Roberta Costi, Francesco Saccoliti

**Affiliations:** 1 Dipartimento di Scienze Farmaceutiche, Università degli Studi di Milano, Via Mangiagalli 25, I-20133 Milano, Italy; 2 Dipartimento di Chimica e Tecnologie del Farmaco, Istituto Pasteur-Fondazione Cenci Bolognetti, “Sapienza” Università di Roma, p.le Aldo Moro 5, I-00185 Rome, Italy; 3 Dipartimento di Scienze della Vita, della Salute e delle Professioni Sanitarie, Università degli Studi “Link Campus University”, Via del Casale di S. Pio V 44, I-00165 Rome, Italy

**Keywords:** photoaffinity labeling, target deconvolution, target identification, antiprotozoal agents, kinetoplastid diseases, *Trypanosoma*, *Leishmania*, malaria, *Plasmodium*, photo-affinity labelling

## Abstract

*Plasmodium*, *Leishmania*, and *Trypanosoma* parasites are responsible for infectious diseases threatening millions of people worldwide. Despite more recent efforts devoted to the search for new antiprotozoal agents, efficacy, safety, and resistance issues still hinder the development of suited therapeutic options. The lack of robustly validated targets and the complexity of parasite’s diseases have made phenotypic screening a preferential drug discovery strategy for the identification of new chemical entities. However, via this approach, no information on biological target(s) and mechanisms of action of compounds are provided. Among the target deconvolution strategies useful to fill this gap, photoaffinity labeling (PAL) has emerged as one of most suited to enable investigation in a complex cellular environment. More recently, PAL has been exploited to unravel the molecular basis of bioactive compounds’ function in live parasites, allowing elucidation of the mechanism of action of both approved drugs and new chemical entities. Besides highlighting new potential drug targets, PAL can provide valuable information on efficacy and liabilities of small molecules at the molecular level, which could be exploited to greatly facilitate the rational optimization of compounds in terms of potency and safety. In this review, we will report the most recent studies that have leveraged PAL to disclose the biological targets and mechanism of action of phenotypically active compounds targeting kinetoplastid diseases (i.e., human African trypanosomiasis, leishmaniasis, and Chagas disease) and malaria. Moreover, we will comment on potential perspectives that this innovative approach can provide in aiding the discovery and development of new antiprotozoal drugs.

## 1. Introduction

Protozoan parasites cause challenging infectious diseases that are responsible for relevant morbidity and mortality globally. Among them, malaria and kinetoplastid diseases (KDs) affect millions of people worldwide and represent a tremendous burden for low- and middle-income countries [1]. Due to their higher prevalence in tropical and sub-tropical areas, they have been historically classified as tropical diseases [2], and, in particular, the World Health Organization (WHO) currently classifies KDs as neglected tropical diseases (NTDs) [3]. However, many factors have recently contributed to the spread of such diseases in non-endemic countries, indicating these as emerging global threats for human health [4,5].

Among the five species causing malaria, *Plasmodium falciparum* (*P. falciparum*) is responsible for most of the severe and deadly forms of the disease, accounting for >90% of malaria mortality globally [6]. The burden of malaria is tremendous: according to WHO, 249 million cases causing 608,000 deaths were estimated in 2022 [7]; indeed, malaria is one of the deadliest parasitic diseases occurring globally, with nearly half of the world’s population at risk for infection [7,8].

KDs are a group of infectious diseases caused by parasites belonging to the Trypanosomatidae family. They include the different forms of leishmaniasis, which are caused by several species of *Leishmania* parasites; Chagas disease (CD), that is due to *Trypanosoma cruzi* infection; and human African trypanosomiasis (HAT), for which the *Trypanosoma brucei* subspecies are responsible [9,10,11,12].

It has been estimated that KDs affect more than 10 million people worldwide, causing nearly 30,000 deaths annually [1,13]. Despite the burden, KDs are still categorized as NTDs, reflecting not only their prevalence in developing countries, but also the low interest of pharmaceutical companies in investing resources for developing therapeutic options for these diseases [14,15,16].

Historically, the search for new antiprotozoal agents has represented a neglected area of research compared to other antimicrobics. Consequently, current antiprotozoal chemotherapy relies mainly on old drugs, whose current use is mainly due to the lack of alternatives, rather than to their effective advantages. Indeed, many of them are far from ideal, suffering from major limits including limited efficacy and relevant toxicity, hampering their use for treating the most challenging and long-term forms of the diseases [1,17,18].

In addition, the onset of resistant parasitic strains is a critical health concern. Prominent examples are the spread of artemisinin-resistant *Plasmodium falciparum* strains in South East Asia and the lack of efficacy of pentavalent antimonials for treating visceral leishmaniasis in the state of Bihar (India) [19,20]. Furthermore, no vaccines are available for KDs, underlining that vaccine development against parasites remains still challenging [1,13,21]. Overall, these findings indicate the imperative need for new therapeutic options relying on the search for more effective and safe antiprotozoal agents.

Fortunately, in the last decades, new research has been performed in the field, highlighting a renewed interest in the search for new antiprotozoal agents. Indeed, both academic and public–private partnerships became more engaged in the antiprotozoal drug discovery pipeline, with non-profit and charitable organizations, such as the Drug Discovery for Neglected Diseases Initiative (DNDi), Wellcome, and the Global Health Innovative Technology Fund (GHIT Fund) [22], providing major contributions and investments to enable the search and discovery of new therapeutic options [1,22,23,24,25]. These efforts have led to the recently approved drug fexinidazole (the first all-oral treatment for the treatment of HAT [26,27,28]) and the identification of new chemical entities, which are currently progressing toward clinical trials for the treatment of malaria and KDs [29,30,31,32].

It is noteworthy that all the antiprotozoal drugs and candidates emerging so far have been identified via the phenotypic approach assessing the compounds’ efficacy to inhibit parasites growth (or induce parasites’ death) in whole-cell screening assays [1,13,16,17,22,33].

On the other hand, despite the attempts, target-based search for antiprotozoal agents demonstrated to be very challenging and had not yielded the expected results so far. Indeed, although progress in molecular biology has enabled the identification and elucidation of some peculiar metabolic pathways of the pathogens, many aspects remain still obscure, and the intrinsic complexity of parasites’ biology is a critical issue [1,13,16,21,22]. Overall, target-based antiprotozoal drug discovery has been hindered by the lack of robustly genetically and chemically validated targets [21,34]. A suited alternative to fill this gap has been provided by high-throughput phenotypic screenings, which have been conveniently employed to screen large libraries of compounds and enable the selection of more promising hit/lead compounds. Indeed, by highlighting phenotypic changes, this strategy takes into account not only the multiple interactions involving disparate targets and pathways in a biological context (pharmacodynamic aspects), but also cellular permeability and related pharmacokinetic issues. Overall, this approach allows bypassing the limits related to a target-based strategy and parasites ’complexity to yield compounds with suited in vitro and/or in vivo efficacy [21,22,35,36,37,38].

Despite these, the phenotypic screening strategy displays liabilities since, indeed, compounds are solely selected based on their effect in whole-cell organisms, but without indications of their biological target(s) and mechanism of action [34,36,39]. Nevertheless, in the context of such a challenging area of research, gaining such information is highly critical to understand the molecular basis of active compounds’ function in whole parasites [34,35].

Elucidation of phenotypically active compounds’ targets can be achieved via target deconvolution (TD) studies requiring additional, though challenging, experiments [34,35,36,38]. Nevertheless, this process is highly attractive in drug discovery and, more specifically, extremely useful for the antiprotozoal drug discovery pipeline according to the following reasons:

(i) TD aids to disclose the molecular basis of phenotypically active compounds’ mechanism of action [35,38]; such strategy can be applied to both investigational compounds and already approved drugs for which the mechanism of action has been poorly understood and/or relies on interaction with multiple targets [1,37,38]. Moreover, this process is key also to elucidate the (usually complex) biological profile of natural sources-derived antiprotozoal agents [40];

(ii) TD enhances development and optimization of phenotypically active compounds: from a medicinal chemistry perspective, deconvolution of drug target(s) for a compound with a demonstrated phenotypic effect could provide useful insights to drive more rational chemical scaffold modifications by aiding in delineating structure–activity relationships (SARs). In particular, it could be of great utility for guiding structural optimization of compounds in terms of efficacy and safety profile [34,35,39,41,42]. Moreover, if the identified target has been previously characterized (i.e., X-ray or Cryo-EM structures are available), such information could be exploited to drive target-based drug design of new analogs;

(iii) Elucidation of drug profiling: identification of targets engaged by antiprotozoal agents could provide important insights about not only the biomolecule responsible for efficacy (on target), but also on those target(s) whose undesired modulation can produce side effects (off target) [22,38,43,44]. Given that toxicity is an important issue for currently employed antiprotozoal drugs, achieving this goal is highly attractive;

(iv) Uncovering still unknown targets or new mechanism of actions: TD has the potential of enlightening biomolecular targets that are not known yet, providing key indications to develop innovative and effective antiprotozoal agents and, in addition, allow more in-depth understanding of parasites‘ biology. Indeed, from a medicinal chemistry perspective, if a particular biomolecule is identified from TD studies, this finding could represent not only a proof-of-concept for target druggability, but also it could indicate the engagement of such a biomolecule in disease pathogenesis [21,22,38]. Thus, if the biomolecule emerging from TD studies was not already known, or if it was not known to be involved in the disease, the TD outcome could greatly aid in the understanding of the pathological basis of the disease. Furthermore, both scenarios could be of critical utility from a drug discovery perspective, leading to the great opportunity to explore “undruggable targets” and develop first-in-class drugs [45]. Indeed, in analogy with (ii), if the structural features of such a biomolecule were known, these could be exploited to pursue a rational and focused target-based drug design.

From the previous points, it is evident that insights derived from TD studies could empower the current antiprotozoal drug discovery pipeline tremendously. Indeed, it could allow filling the gaps left by either phenotypic- and target-based drug discovery strategies to enhance parasitic diseases’ comprehension and drive the development of more effective and safe antiprotozoal drugs [38].

Owing to the complexity of parasites ‘cellular biology, TD studies should be performed in a way to furnish reliable information about the biological behavior of the phenotypically active compound. In other words, experiments should be performed in a native cellular environment (i.e., live cell parasites) in order to resemble, as much as possible, the experimental conditions set in phenotypic studies and avoid misleading results.

Among the suited TD strategies, photoaffinity labeling (PAL) emerged recently as one of the most promising. Indeed, besides its various applications, PAL has been exploited as a powerful chemo-proteomic approach to profile the target landscape and unravel the mechanisms of action of bioactive compounds in live organisms [38,46,47,48].

Interestingly, PAL has also been recently applied in the antiprotozoal drug discovery field, and TD studies have been performed to elucidate the mechanism of action of both already approved drugs and experimental compounds. These findings, which notably further stress a renewed interest in the field, are of valuable interest, bearing the potential to enhance in-depth understanding of the current state-of-the art and facilitate the advances in the discovery of innovative antiprotozoal agents.

In this review, we will report and comment on the most recent applications of PAL for TD purposes in antiprotozoal drug discovery, with a major focus on antimalarial and anti-kinetoplastid agents and drugs. In this scenario, this review aims to describe the PAL technique and the rational basis useful for designing useful chemical probes to be employed in these experiments. Furthermore, a collection of PAL experiments employed in the field of KDs (i.e., HAT, leishmaniasis, and CD) and malaria for the identification of the mechanism of actions underlying the effect of new chemical entities and already approved drugs are herein described.

From a literature survey, it can be noticed that most research articles have focused on malaria, reflecting the higher interest and global distribution of the disease. On the other hand, an increasing number of studies are applying PAL also to KDs, with some of them focusing on a single parasite (mainly *T. brucei*), while others refer to various trypanosomatids. Notably, the latter approach is particularly attractive and has the potential to enlighten druggable target(s) of different kinetoplastids for enhancing the development of broad-spectrum antiprotozoal agents.

By providing the most recent insights in the field, we aim not only to collect the most recent research in the field, but also to stimulate new ideas and efforts for advancing the search for innovative therapeutic agents.

## 2. Photoaffinity Labeling (PAL)

In recent years, PAL has emerged as a powerful chemo-proteomic approach to disclose the molecular basis of bioactive compounds’ function in cellular disease models. In particular, PAL can be used to elucidate the target profile of bioactive compounds, and consequently elucidate their mechanism of action, in live cells [38,46,47,48]. Thus, it is a suited TD strategy for identifying molecular targets that underlie the phenotypic effects of bioactive compounds, including drugs.

More in general, PAL can be employed for comprehensive drug profiling, enhancing the detection of either on and off targets, and providing insightful indications to understand both bioactivity and toxicity in intact organisms. In addition, PAL is a powerful and straightforward strategy to elucidate an active compound binding via ligand-binding site mapping. Apart from drug discovery purposes, PAL has been also applied to map interactions occurring in the biological environment, allowing the in-depth elucidation of still poorly understood biological processes occurring in either physiological and pathological conditions [48,49,50]. According to these, PAL has emerged as an important chemical biology tool with interdisciplinary applications that could importantly enhance both knowledge of the disease and the drug discovery pipeline.

For TD purposes, PAL relies on the use of chemical probes structurally related to the phenotypically active compound to engage the same target as the parent compound in the native environment. However, since drug-target binding occurring in a biological context usually relies on reversible (non-covalent) and transient interactions, an additional step is required to allow the stable cross-linking of the engaged target biomolecule(s) and enhance the next proteomic analysis. This is made possible by the inclusion of a photoreactive group (PG) on the scaffold of the parent compound, whose activation by UV wavelengths is responsible for turning such reversible interactions into irreversible ones. The resulting probe–biomolecule adducts can be then analyzed to identify the target (target ID) via biochemical, molecular biology experiments (e.g., Western blot, in-gel fluorescence, and imaging) or analytical techniques (i.e., LC-MS/MS analysis) [38,46,47,48,51].

**Figure 1:**
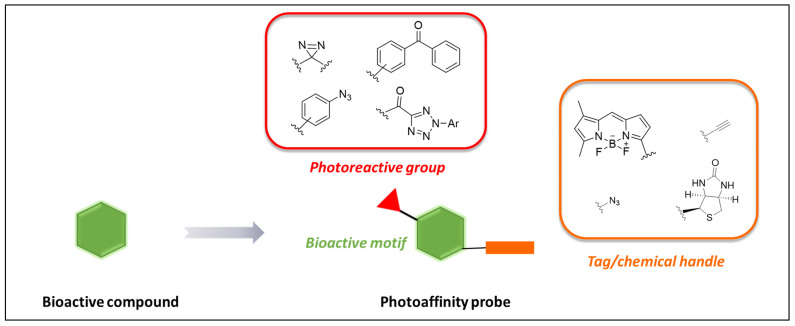
Main structural and functional features of a photoaffinity probe.

Photoaffinity probes (Figure 1) are endowed with the following features:

(i) a bioactive motif that is basically the pharmacophore scaffold of the parent compound, to drive activity and selectivity toward target biomolecule(s) and mimic the biological profile of the parent [48,51];

(ii) a PG inserted or appended to the main scaffold of the parent compound (bioactive motif). UV-induced photoactivation of such a moiety produces a highly reactive species capable of cross-linking nearby biomolecules and, thus, enabling capturing of engaged target(s) via formation of stable probe–biomolecule adducts. PGs are chemical groups with “dormant” reactivity that are activated only in response to UV irradiation. Indeed, to be suited for PAL studies, PGs must be stable in physiological conditions and daylight. In addition, they should activate at UV wavelengths not detrimental for biological systems (ideally with λ > 300 nm) to minimize interference on concomitant cellular processes and preserve native conformation target biomolecules [48,50,51,52];

(iii) a reporter/purification tag, to allow the detection, identification, and characterization of the generated adducts. Despite not being involved neither in the engagement nor in the capturing of the target biomolecule(s), such group plays a key role in facilitating the target ID [48,51].

Indeed, when PAL studies are performed in a complex cellular environment (i.e., live cells), a tag is critical to enhance detection and/or isolation of the labeled adducts from the resting and crowded proteome.

Depending on the purpose of the study, different tags can be employed, in an exclusive or complementary way. Common tags include a fluorophore or dyes (suitable for in-gel fluorescence and bioimaging studies), or a biotin moiety (for target enrichment via streptavidin/avidin pull-down), or radioligand suitable for radio imaging studies [49].

A tag can be inserted on the structure of the probe either directly, that is, pre-installing the tag on the structure of the probe, or indirectly via a chemical handle suitable for conjugation to a tag reagent via bioorthogonal ligations [48,49,53]. However, the latter strategy became very popular in recent years and has been widely employed in PAL studies. Indeed, by allowing probe–tag ligation in situ, it permits the incubation of smaller probes, which are more likely to mimic the biological behavior of the parent [48,49,52]. Among the strategies exploited for conjugating the probe and tag reagent, copper-catalyzed alkyne-azide cycloaddition (CuAAC) [54,55,56] is the most popular [51,52,53]. However, owing to the toxicity of copper for live cells [57,58], cells are usually lysed upon performing in situ tagging.

In a typical PAL experiment, a probe is incubated with live cells to engage its target(s). UV photoactivation allows the capturing of engaged target(s) to form probe–biomolecule adducts. If a tag has been pre-installed on the probe, the properties of such a moiety (i.e., fluorescence, affinity toward streptavidin or antibody) can be exploited to discriminate or isolate the adduct from the resting proteome. On the other hand, if a chemical handle is present (e.g., alkyne click handle), a tag reagent with complementary reactivity (e.g., azide group) can be added to in situ label the adduct (e.g., via CuAAC) and, thus, enhance their detection and identification via biochemical techniques and/or LC-MS/MS. In the latter case, proteolytic treatment is required to induce proteolytic digestion to provide shorter peptide–probe adducts, which can be analyzed via LC/MS-MS analysis in quantitative proteomic analysis. Notably, the insertion of an analytical handle, that is, a chemical moiety bearing a peculiar isotopic pattern, can greatly facilitate the target ID via LC/MS-MS experiments. Besides representing an alternative or complementary strategy to detect adducts from a complex biological mixture, LC-MS/MS analysis is also a powerful tool to map a ligand-binding site. Extrapolating such information is highly important from a drug discovery perspective, since it allows detecting the structural features involved in ligand binding in a native environment, providing valuable insights to guide target-based drug design [48,52,59,60].

Besides performing PAL studies in live cells being highly attractive, performing these investigations in such a complex cellular environment can, to some extent, furnish misleading results, usually resulting from non-specific labeling events. Thus, in the context of TD studies, it is critical to confirm that the biomolecules labeled by the probe experiment are also actual target(s) for the parent.

**Figure 2:**
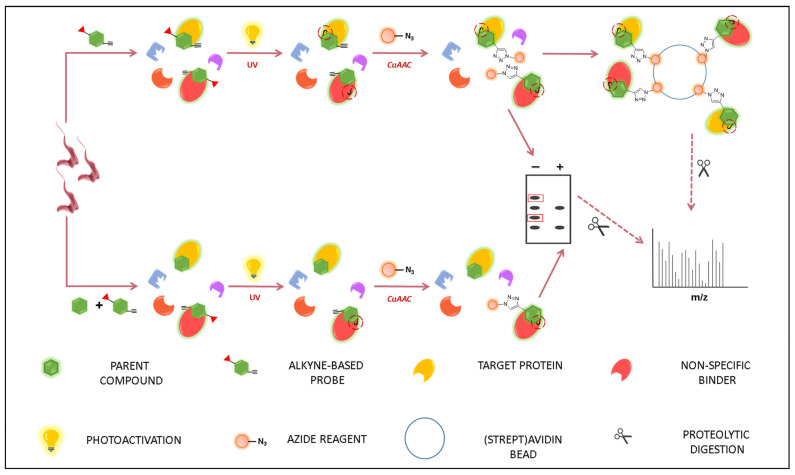
General PAL workflow (upper flow) and competition assay (lower flow) relying on in situ tagging, affinity purification, electrophoresis, and analytical analyses to disclose specific and non-specific binders of alkyne-based probes for target ID.

A common experiment to confirm this finding is competition assay, in which an excess of the parent compound is incubated with live cells prior to probe incubation. Indeed, owing to the higher affinity of the parent toward its target(s) than the probe, pre-treatment with parent compound led to saturation binding, which, overall, hinders the binding of the probe. Consequently, by comparing data from experiments performed with a probe only and the ones relying on parent and probe together, indications are provided on the actual target for the parent. At the same time, such a strategy allows detecting a false-positive resulting from non-specific labeling of the probe to close biomolecules. For instance, the disappearance of protein bands previously detected by gel electrophoresis analysis confirms that such bands are referred to target(s) for the parent, while, conversely, their persistence indicate that these are targeted by probe only, and, indeed, are referred to non-specific labeling events (Figure 2) [48,49,60].

From the previous points, it is evident that PAL is a powerful technique to provide a comprehensive target profile for a bioactive compound in live cells. According to its set-up, PAL represents a suited target-fishing strategy, since it allows engaging, capturing, and characterizing biomolecular interactions of a certain compound by using its structurally related probe [51,59].

Despite its potential as a powerful TD strategy, PAL displays also intrinsic liabilities, whose knowledge of and careful evaluation is key to address the set goal and limit possible misleading results. Non-specific labeling of several biomolecules that are not the intended targets is a common feature of PAL experiments that is mainly due to the formation of highly reactive species (i.e., carbenes, radicals) resulting from UV-photoactivation. This event is particularly relevant in live-cell experiments and can greatly increase data complexity. More often, the probes cross-link to highly abundant or concentrated proteins of the sample. In addition, the physical–chemical properties of the probe can impact labeling preferences via electrostatic or hydrophobic interactions. Thus, to facilitate data interpretation, additional assays need to be performed (e.g., competition assays and/or experiment with negative controls). According to this, PAL is not a stand-alone technique, and photoactivation experiments need to be integrated with additional and complementary biochemical and analytical experiments (e.g., Western blot analyses, LC-MS/MS analyses) to confirm the engagement of the intended target [51,52]. However, performing such additional assays, together with data processing and analysis, can be complex and time- and resource-consuming. In addition, the experimental set-up is key though challenging, since each step of the PAL workflow requires optimization (e.g., choice of probe concentration, duration and conditions of photoactivation, conditions of washing and elution) to avoid artifacts. Moreover, highly reactive intermediates that formed upon photoactivation are quickly quenched by the cellular medium [48,49,50,51,52]. Despite such an event being able to minimize non-specific binding, it can also be disadvantageous by lowering cross-linking yields, that, for instance, have been estimated to be under 10% for a diazirine-based probe [51,52]. This issue can render difficult the identification of lower abundance proteins, and, conversely, increase the labeling of more abundant or highly concentrated biomolecules. In light of such a limitation, the employment of a PAL probe with very high affinity toward the target is critical to increase, as much as possible, the yield of photoactivation and reduce a non-specific background [48,49,50,51,52]. According to this, the probes‘ design is key, since the choice of the suited groups to be attached to the bioactive motif, as well as the design strategy adopted, can greatly impact the PAL yield. On the other hand, several rounds of synthetic efforts and biological evaluations could be needed to achieve this goal.

Thus, despite being essential, the search for the “ideal” probe could be a long and time- and resource-consuming process, relying on multiple interdisciplinary and iterative rounds of optimization.

### Probes’ Design Considerations

The first and critical step of the PAL workflow is the design of the probe, which needs to take into account both medicinal chemistry and chemical biology aspects. Indeed, probe design can importantly affect all the downstream steps of the PAL strategy, since a suited design approach can more likely enhance the achievement of the set goal, while, on the contrary, a more random strategy could yield misleading results [38,52,59].

However, it is important to underline that there is not a unique, universal, and valid strategy for probes ‘design: it is strictly dependent on the specific compound (and its structure), upon which TD studies are relying. The chemical structure of the bioactive compound is a strong basis on which a probe can be built; however, chemical manipulations are needed to insert the two key groups (PG and tag/chemical handle) essential for PAL studies.

First of all, it is central to recognize which parts of the parent scaffold do not affect critically the bioactivity and distinguish them from those points that instead do greatly. Indeed, the former should not be involved in target(s) modulation and could be chemically modified for inserting the key groups. Differently, the chemical portion affecting bioactivity needs to be left untouched. Indeed, this moiety, that is, the pharmacophore, needs to be maintained as such also in the probe, since, by acting as a bioactive motif, it will engage the same target as the parent. On the contrary, modification of such a group could importantly affect the biological profile of the probe, leading, for instance, to the engagement of a different biomolecule, yielding, overall, to the deconvolution of a false target and thus misleading results [38,60].

Identification of pharmacophoric and non-pharmacophoric (accessorial) moieties is enabled by the generation of a set of structurally related analogs and their evaluation in phenotypic assays. Indeed, deep knowledge of structure–activity relationships (SARs) of the parent compound greatly facilitates and drives the design of probes since the parent scaffold should be modified accordingly. According to this, SARs can provide more direct indications of points amenable for chemical modifications for inserting key groups of the probe.

However, besides bioactivity, the physical–chemical properties of the parent compound should be also taken into account. To mimic the parent in terms of both target engagement and cell permeability, chemical manipulations should be applied in such a way to produce minimal perturbation of the physical–chemical profile of the parent compound. Indeed, since probes are usually incubated in live cells, requiring crossing multiple barriers to reach the target, this is a critical issue to be considered.

Apart from the bioactive motif, the choice of the PG and tag/chemical handle is equally critical. In both cases, steric and physical–chemical properties of these groups need to be carefully considered, since the insertion of highly sterically demanding groups, or moieties affecting the polarity of the parent, could importantly impact on either target binding and the cellular permeability of the probe [38,51].

Concerning the PG, its reactivity, and steric hindrance are critical aspects to be considered. Most commonly employed PG groups include arylazides, benzophenones, and diazirines, each showing different reactivity and steric properties. All PGs employed in PAL are UV-activated to highly reactive species (carbenes or radicals), which are critical to quickly capture close biomolecules and avoid their diffusion away from the binding site. However, while arylazides require more energetic UV wavelengths, benzophenones and diazirines are activated by longer UV wavelengths, which are more suited to concomitant biological processes, rendering such PGs more preferred for PAL investigations. Despite this, steric properties of PGs need to be taken into account, since more sterically demanding groups could importantly impact on binding by hindering target engagement via the bioactive motif. Indeed, owing to the higher steric hindrance of benzophenones compared with diazirines, the latter are generally preferred in PAL studies and became very popular in recent years. In particular, among diazirine PGs, the aliphatic diazirine are even more preferred as a low sterically demanding PG. In addition, differently from benzophenone and aryldiazirine, inclusion of such a small PG on the parent scaffold should not dramatically impact physical–chemical properties of the parent [47,48,49,50,51,52].

Another critical aspect to be considered in probe design is the insertion strategy applied to introduce the key groups. In general, such moieties could be inserted on the parent scaffold either directly or via a linker moiety. Direct insertion of the PG could be envisaged as a suited strategy to place such a group in close proximity to the engaged target to enhance its capturing. However, although the target proximity could minimize non-specific labeling, too close insertion of the PG could sterically hinder target engagement [49,60].

On the other hand, if a connector is inserted, the length and nature of such a linker are critical since such modification can affect both target recruitment and physical–chemical properties of the probe. Indeed, if a linker is used to connect the PG with the bioactive motif, the length of such a connector should be carefully evaluated, since too long linker could address the PG far away from engaged target. This can led to non-specific labeling, since the PG could capture target(s) close to it rather than those actually engaged by the parent scaffold [49].

Although the choice is strictly dependent on the specific case, the use of a shorter linker to connect both the PG and the tag/handle to the bioactive motif is preferred. According to this, a “minimalist” linker bearing both key groups along a short chain represents a convenient and very popular option. This “all-in-one” strategy is advantageous since it allows inserting both key groups in a single point of the molecule, minimizing both chemical manipulation of the parent scaffold and synthetic efforts required to access the probe [58,61].

Overall, all these aspects need to be carefully considered in designing a probe. In general, since little modifications could impact biological activity importantly, it is more convenient to synthesize a small set of probes (rather than only one) via some of the alternative strategies herein described [52].

Importantly, to select the best candidate(s) for PAL studies, biological activity check via phenotype-based assay is strictly required: only probes showing an activity profile comparable to the parent are suitable for PAL experiments. Indeed, broad activity discrepancies between probes and parent may indicate a divergent biological profile or could suggest differences in their physical–chemical properties. Conversely, only probes showing phenotypic activities comparable with the parent (within the same order of magnitude of the concentration range) are more likely to mimic its biological profile, and should be preferentially selected as suitable tools to perform TD studies.

## 3. Kinetoplastid Diseases (KDs) and Their Therapeutic Options

Protozoan parasites that cause Kinetoplastid diseases are a group of NTDs caused by species of *Leishmania* and *Trypanosoma* parasites, which threaten millions of people worldwide. They are mainly spread in developing countries, where they represent important causes of morbidity and mortality among the world’s poorest populations. They encompass Chagas disease (CD), different forms of leishmaniasis, and Human African Trypanosomiasis (HAT), each showing a different distribution worldwide [9,10,11,12,62,63,64]. Although most cases occur in impoverished countries with poor health resources, recently some KDs have broadened their frontiers worldwide. Climate changes, vector and human migrations, organ transplant/blood bank contaminations, and co-infection with HIV are some of the factors contributing to such global spread of the disease [5,21,65,66,67].

Parasites are dixenic, cycling between different invertebrate vectors and mammalian hosts, showing substantial differences in morphology, cell biology, and biochemistry between life cycle stages, and, in some cases, between species [9,21].

Vaccine development is a powerful approach to disease management but remains challenging in this area, due to efficient immune-evasion mechanisms or the intracellular locations of parasites in the host [1,21,68].

On the other hand, chemotherapy for KDs is unsatisfactory, relying on mainly old drugs that probably act on multiple parasite targets and have several modes of action, which contribute to their relevant toxicity [1,13,37]. In addition, despite being generally competent in curing acute and milder conditions, they usually fail to contrast chronic and more challenging stages and are not potent enough to produce a sterile cure [1,13]. Moreover, many of them require parenteral administration, presenting a logistical challenge, especially in resource-poor rural settings where they are endemic [21,69].

CD is caused by *Trypanosoma cruzi* (*T. cruzi*), which is transmitted to humans by contact with the feces or urine of triatomine bugs (kissing bugs), despite alternative transmission routes having been reported that are likely to occur in non-endemic settings. The disease is endemic in Latin America, where 6–8 million people are infected, while 75 million are at risk of contracting the disease. However, in the last decade, CD has been found also in other countries, including Europe and USA [13,21,63,67,70].

CD is characterized by an acute stage, in which mild or vague symptoms can occur, followed by a chronic stage marked by low parasitemia. In most cases, no disease occurs throughout life; however, in nearly 30% of infected patients, symptomatic chronic symptoms can occur within 10–30 years. The heart and gastrointestinal tract are particularly affected, and in particular, cardiopathies are the most prevalent and life-threatening manifestations [63,71]. The pathogenesis of the chronic CD is not fully understood [1,13], rendering it very challenging to find a sterile cure. Growing evidence indicated the existence of *T. cruzi* persisters [72], which could account for the failure of drugs in producing complete eradication of the chronic parasite infection [1].

**Figure 3:**
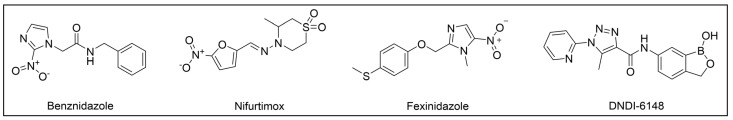
Chemical structures of antichagasic drugs (i.e., benznidazole and nifurtimox) and chemical entities in development for CD (i.e., fexinidazole and DNDI-6148).

Nitroheterocycles nifurtimox and benznidazole (Figure 3) are the sole therapeutic options available for CD since 1960s [73]. While both drugs proved to be effective against acute infection, their efficacy toward chronic CD is still debated [1,13,74]. Moreover, both drugs display serious side effects, which can be responsible for treatment discontinuation [13,69,74] and are contraindicated in pregnancy.

Owing to the still obscure pathology of CD, the target-based search for new potential antichagasic drugs has been limited; despite this, some recent efforts were also focused on parasite enzymes [74], such as enolase [75], cruzain [76], ribose-5-phosphate isomerase (RPI) [77,78], isocitrate dehydrogenase (IDH2) [79], sirtuins [80,81,82], and sterol 14α-demethylase (CYP51) [83,84,85]. However, owing to the recent failure of posaconacole, ravuconazole, and fosravuconazole [86,87,88,89], inhibitors of the latter enzymes have been de-prioritized in drug discovery programs [1,90].

The still incomplete understanding of CD, and the great challenges in identifying molecules capable of producing complete parasite eradication, is reflected in the poor drug discovery and development pipeline for such disease [1,13,91]. Nowadays, only the already approved anti-HAT drug fexinidazole (Figure 3) is currently in phase II for CD, while the development of the oxoborole DNDI-6148 (Figure 3), which has been evaluated also for the treatment of leishmaniasis, has been paused pending further studies to determine the potential for reproductive toxicity [1,92,93,94].

Leishmaniasis is caused by over 20 species of *Leishmania* parasites and displays broader global distribution than CD and HAT. Indeed, it is most prevalent in Latin America, South East Asia, East Africa, and North Africa, where nearly one billion individuals are at risk of contracting the disease [64]. It has been estimated that 700 thousand–1 million new cases occur annually, and the disease is responsible for 26,000–65,000 fatalities [64,68,95], rendering leishmaniasis the second biggest parasitic killer in the world, after malaria [68].

*Leishmania* is transmitted by the bite of infected sandflies and can cause three different main diseases: visceral leishmaniasis (VL, or kala-azar), cutaneous leishmaniasis (CL), and mucocutaneous leishmaniasis (MCL). CL is responsible for self-healing or permanent skin scars, while MCL leads to partial or total destruction of mucous membranes of the nose, mouth, and throat, causing disfiguring lesions of the face. Among them, VL is the most clinically relevant form of leishmaniasis, since it is almost fatal if left untreated. VL, which is caused by *Leishmania infantum* (*L. infantum*) and *L. donovani*, is a systemic infection causing fever and weight loss and affecting many organs, including the spleen, the liver, and the bone marrow [19,64,68]. In addition, co-infection with HIV is an important concern, since it accelerates the progression of both diseases and, in addition, has been responsible for the re-emergence of VL in Southern Europe in the late 1990s [19,21,65,66].

**Figure 4:**
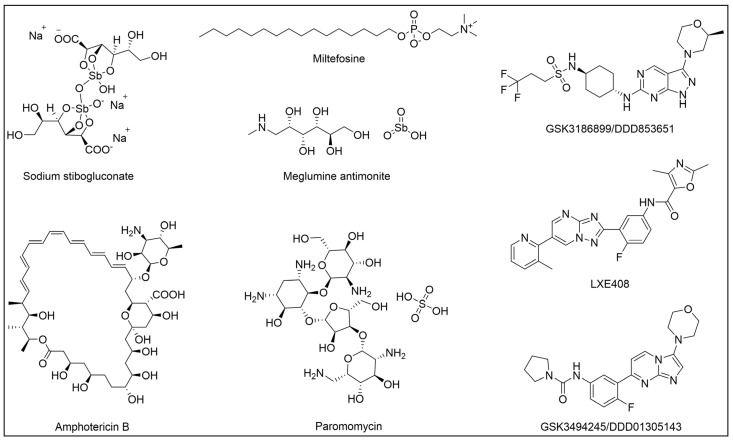
Chemical structures of antileishmanial drugs (i.e., sodium stibogluconate, amphotericin B, miltefosine, meglumine antimonite, and paromycin) and chemical entities in development for VL (i.e., GSK3186899, LXE408, and GSK3494245).

Most of the available drugs have been repurposed from other indications. The therapeutic scheme is dependent on the form of the disease, parasite species, immune status of the patient, geographic location, and, in turn, availability of drugs and their cost on that region [19,68,74]. Antimonial drugs have been used for the treatment of leishmaniasis since the 1900s [96] and have represented the first-line option for many decades. Sodium stibogluconate and meglumine antimonite are the pentavalent antimonial drugs employed so far (Figure 4). The proven effectiveness of such drugs against *Leishmania* parasites has prompted their long-term use as standard therapies for the treatment of different forms of leishmaniasis. Moreover, clinical experience has provided a solid understanding of the drugs’ efficacy and dosing, and, according to this, antimonial drugs have been approved and widely accessible in endemic regions. Furthermore, in areas with limited healthcare infrastructure, antimonials are often more cost-effective than newer treatments, making such drugs preferred therapeutic options in resource-limited settings. Despite these, the pharmacological profile of such drugs is far from ideal, and their use suffers from major drawbacks. Indeed, they require long-term intravenous or intramuscular administration, and cause potentially life-threatening side effects, including cardiotoxicity, hepatotoxicity, and pancreatitis. Nowadays, the efficacy of such drugs has proven to be highly dependent on the region, and, indeed, high rates of resistance are observed in many regions of India [19,69]. Liposomal formulations of the antifungal drug amphotericin B (Figure 4) are a suited alternative in cases of antimonials failure; however, although such formulation can partially mitigate the toxicity of the drug, administration requiring trained healthcare professionals and its high cost are important limitations in endemic countries [68,74].

The aminoglycoside paromomycin (Figure 4) is a further therapeutic option; it is available from the 1980s and is generally employed as a second-line drug and in a combination regimen with others antileishmanial because of the risk of drug resistance [74,97].

Miltefosine (Figure 4) is an anticancer drug that has been repurposed more recently for the treatment of leishmaniasis; it is the only oral antileishmanial drug approved so far. The drug is recommended in combination with other drugs to increase the therapeutic efficacy, shorten the duration of therapy, and avoid drug resistance. However, the teratogenic effect of this drug is an important limit that hinders its use in pregnancy [74].

In recent years, some efforts have been devoted to the search for antileishmanial agents capable of modulating various parasitic metabolisms [74,97], including purine salvage pathway [98,99,100], mitochondrial electron chain and cytochromes [101,102], redox metabolism [18,103,104,105,106], folate metabolism [107,108,109,110], and protein kinases [111,112].

Compared to CD, the drug discovery pipeline for leishmaniasis proved more promising. Most efforts were focused on VL and led to the identification of new chemical entities, some of which are currently under investigation in clinical trials [1,68]. Although most of these compounds were identified by the phenotypic approach, target deconvolution studies were also performed to elucidate their mechanism of action [113,114,115,116]. They include GSK3186899/DDD853651 [32,117] that is currently in a phase I clinical trial and proved to inhibit cdc2-related kinase 12 (CRK12) [116], while GSK3494245/DDD01305143 (phase I) [115,118,119] and LXE408 (phase II) [113,120] resulted in being proteasome inhibitors (Figure 4). On the other hand, toxicity liabilities were highlighted for oxaborole DNDI-6148 [93,121] and nitroimidazole DNDI-0690 [122].

HAT is caused by the two *Trypanosoma brucei* (*T. b.*) subspecies *T. b. gambiense* and *T. b. rhodesiense*, which are transmitted to humans by the bite of an infected tsetse fly, although animals can represent an important reservoir for *T. b*. Differently, *T. brucei brucei* is the causative agent of animal trypanosomiasis (Nagana disease). *T. b. gambiense* causes 98% of HAT cases and is endemic in West and Central sub-Saharan Africa, while *T. b. rhodesiense* is responsible for a less common (but more aggressive) form of HAT in East and Southeast sub-Saharan Africa [62,123]. Fortunately, huge efforts devoted by WHO according to the set road map has made the elimination of this disease a realistic target by 2030. Indeed, fewer than 1000 cases per year were reported for the fifth year in a row; that is a great achievement in light of the 35,000 cases reported per year two decades earlier [62,124,125].

HAT comprises two phases: in the first hemolymphatic stage, parasites localize in the blood and are responsible for vague symptoms including intermittent fever and headache, while the second (meningoencephalitic) stage is most challenging and is characterized by invasion of the central nervous system. Indeed, in this phase, main symptoms, including sleeping and neuropsychiatric disorders, become evident and lead to coma and death in the absence of treatment. *T. b. rhodesiense* HAT is typically more aggressive, since progression from the first- to the second-stage disease occurs more rapidly (a few weeks or months) than in *T. b. gambiense* HAT [62,123,126].

**Figure 5:**
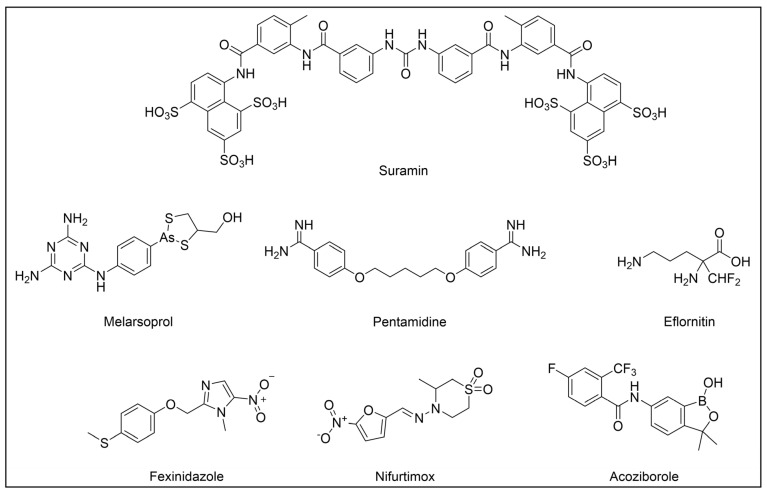
Chemical structures of anti-HAT drugs and acoziborole, the new chemical entity in development for HAT.

Anti-HAT drugs include mostly old drugs used to treat different stages or forms of the disease. Therapy for *T. b. gambiense* HAT has relied on pentamidine (for the first stage) and eflorinitine (for the second stage), with the latter being replaced by a combination of nifurtimox–eflorinitine (NECT) in recent years (Figure 5). However, all these drugs display liabilities mainly related to low-compliance administration routes and important side effects [1,21,74,127]. In this context, the recently approved drug fexinidazole (Figure 5) represents an important milestone for the treatment of *T. b. gambiense* HAT, as the first all-oral drug approved for the treatment of both stages of such disease. According to these, fexinidazole is now the recommended option for the treatment of *T. b. gambiense* HAT [27,28,128].

On the other hand, treatment of *T. b. rhodesiense* HAT has relied on very old drugs, including suramin, for the first-stage treatment, and melarsaprol for the second stage (Figure 5). However, both drugs require intravenous administration and cause serious side effects; in particular, adverse reactions to the organoarsenical drug melarsaprol are common and can be severe or even life-threatening, since a potentially fatal reactive encephalopathy [1,21,74] can occur in up to 10% of cases.

Very recently, EMA provided a positive opinion for the use of fexinidazole also for the treatment of both stages of *T. b. rhodesiense* HAT [129,130], and the drug has been included for such indication in WHO guidelines [26,131]. Overall, the discovery of fexinidazole has revolutionized the therapy for HAT, paving the way for the achievable eradication of such disease.

Despite the approval of fexinidazole being a major step forward in the treatment of HAT, there are still some challenges associated with its use that are deemed to be considered. Common side effects with the drug (e.g., vomiting, nausea, tremors, dizziness) are generally mild to moderate, but can reduce patient compliance. Moreover, neuropsychiatric symptoms (e.g., anxiety, insomnia, depression) can be experienced, rendering the treatment of patients with advanced stages of HAT complicated. In addition, serious side effects of fexinidazole include hepatotoxicity, neutropenia, and cardiotoxicity (i.e., QT interval prolongation), which impede the use of such drug in patients with liver, blood, and heart diseases, or in those assuming concomitant drugs with similar side effects. According to these, careful monitoring of liver, bone marrow, and heart functionality is needed throughout the therapy, which in turn requires healthcare and resources that are usually limited in endemic countries. In addition, the use of the drug is not recommended in children under 6 years of age and in pregnant woman [132]. From a logistical point of view, the collaboration and commitment of international organizations like WHO and local governments are imperative to ensure the availability and affordability of fexinidazole in all the endemic countries over the next years. According to these, despite the huge work carried out by DNDi and its partners so far, increasing and growing efforts are continually needed to ensure healthcare literacy, the equipment of health centers, and the training of healthcare personnel on drug administration and pharmacovigilance in endemic regions. Moreover, despite being only recently approved, it is likely that its widespread use could facilitate the onset of drug-resistant strains in the next years, underlining the importance of identifying further drug alternatives. In this scenario, the development of the orally active oxaborole acoziborole (Figure 5), a clinical entity currently undergoing advanced phase IIb/phase III clinical studies for HAT treatment, is highly promising [133].

## 4. PAL Studies on KDs

### 4.1. PAL Studies on Trypanosoma Brucei

**Figure 6:**
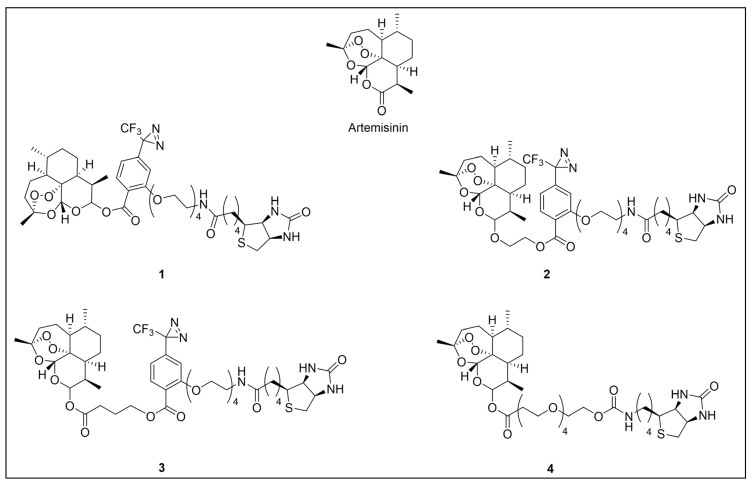
Chemical structures of artemisinin and artemisin-based probes **1**–**4**.

Besides being an antimalarial drug, artemisinin also possesses antitrypanosomal activity [134,135]. Although its target and mechanism of action have been elucidated in *P. falciparum* [136,137], no similar investigations have been performed in *T. b. brucei*. To unravel the target receptor proteins of artemisinin in *T. b. brucei*, a PAL strategy was applied [138]. To this purpose, a set of four probes structurally related to artemisinin were designed and synthesized (Figure 6) [139]. In particular, a dihydroartemisinin scaffold, that is the bioactive form of artemisinin, has been linked to a trifluoromethylphenyl diazirine moiety [140] either directly (probe **1**) or, alternatively, via an ethylene (probe **2**) or butyryl spacers (probe **3**). In particular, such linkers were introduced not only to mimic the artemisinin structural analogs artemether and artesunate, but also to minimize a possible steric clash resulting from the excessive proximity of PG to the bioactive motif that could hinder the binding of the ligand to its target. In addition, since a highly reactive free radical species might result from catalyst-dependent heterolytic cleavage of the artemisinin endoperoxide bridge, as observed in *P. falciparum* [141], a probe lacking the diazirine PG (probe **4**) was also synthesized as a potential activity-based probe. All probes were endowed with a biotin tag to enhance the detection and isolation of labeled proteins by exploiting the strong biotin–streptavidin interaction.

Although trypanocidal activities of the probes were not reported, the author stated that all four probes retained to a certain extent the trypanocidal property of artemisinin. Interestingly, all probes showed good uptakes within parasites (81 to 96% after incubations of only 30 min), indicating that modifications applied in probes did not affect their capability to diffuse within the parasitic cell [139].

In PAL experiments, each probe was incubated with *T. b. brucei* cells, and the preparation underwent photoactivation and subsequent cellular lysis. Obtained cellular lysates were subjected to target enrichment and Western blot analysis for adducts detection by using the horseradish peroxidase (HRP)-tagged streptavidin–biotin method. In all experiments with diazirine-bearing probes (**1**–**3**), a 66-kDa protein band was detected. However, the intensity of such a band was not affected in competition assays, indicating that artemisinin and such probes did not compete for the same target protein. This finding suggests that the detected band could be due to non-specific protein labeling, which could result from the high reactivity of in situ activated diazirine. At the same time, this outcome suggests that the insertion of the bulky aryl diazirine PG close to the dihydroartemisinin scaffold could greatly influence and hinder the binding of such a bioactive motif to its biological target. Conversely, experiments performed with the diazirine-lacking probe (**4**) highlighted three main protein bands at 60, 40, and 39 kDa, which, notably, disappeared in the competition assay, indicating that they are targets for artemisinin. Interestingly, this data confirmed the initial assumption, according to which also in *T. brucei*, an enzymatic activation of the endoperoxide bridge can occur to yield a reactive radical warhead, resembling a mechanism already described in *Plasmodium*. No further studies were performed to elucidate the identity of such protein bands, even though extrapolating such information could provide important insights for the rational design of more potent artemisinin structural analogs endowed ideally with antitrypanosomal and antiplasmodial activities [139].

**Figure 7:**
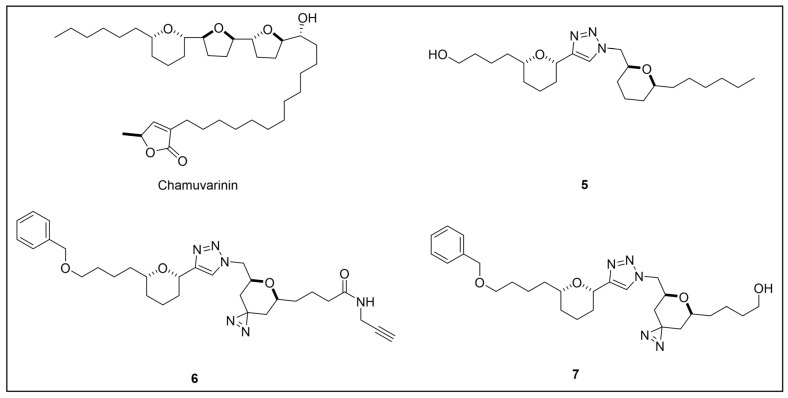
Chemical structures of chamuvarinin, B-THP-T derivative **5**, and related probes **6** and **7**.

Acetogenins are a family of natural products endowed with fatty acid-like structures that have been isolated from tropical plants of the *Annonaceae* family [142]. These natural compounds show peculiar tetrahydropyran (THP) and/or tetrahydrofuran (THF) rings bearing a terminal γ-lactone moiety and a hydrophobic tail. Many acetogenins showed antitumoral properties that have been ascribed to the inhibition of mitochondrial complex I [143,144,145], for which SAR studies indicated the critical role of THP/THF and γ-lactone moieties [146,147]. Notably, chamuvarinin and non-natural bis-tetrahydropyran 1,4-triazole (B-THP-T) analogs (Figure 7) demonstrated antitrypanosomal efficacy against both procyclic and bloodstream forms of *T. b. brucei*, with EC_50_ values within the low micromolar range [148,149], as exemplified by compound **5** (EC_50_ vs. procyclic *T. b. brucei* = 8.4 µM; EC_50_ vs. bloodstream *T. b. brucei* = 1.8 µM) (please refer to Table S1 for activity data) [149].

However, since mitochondrial complex I has been demonstrated to be not essential for both forms of the parasite [150,151], and, additionally, B-THP-T analogs lack the (anticancer critical) γ-lactone moiety, which proved to be key for mitochondrial complex I inhibition, alternative target(s) accounting for a different mechanism of action should occur in the parasite. To perform such investigation, two probes structurally related to compound **5** were designed and synthesized to be employed in PAL studies (Figure 7) [152]. In particular, a bi-functional probe **6** bearing a diazirine PG and an alkyne click handle in different parts of the molecule was designed and synthesized; in addition, a diazirine probe **7** lacking the click handle was used as a negative control. The probes were evaluated for their phenotypic effect on both procyclcic and bloodstream forms of *T. b. brucei* and, in addition, for their cytotoxicity against HeLa and Vero mammalian cells. Both probes resulted in being nearly seven times less active than the parent against the bloodstream form of *T. b. brucei* (EC_50_ (**6**) = 13.0 µM; EC_50_ (**7**) = 11.8 µM); on the other hand, **7** showed similar anti-*T. b. brucei* activity as the parent against the procyclic form of the parasite (EC_50_ = 8.9 µM), while **6** resulted in being nearly two times less active (EC_50_ = 16.1 µM). Overall, the probes showed similar antitrypanosomal activities to **5**, indicating that such modifications did not dramatically affect their biological profile and suggesting that the probes should engage the same target(s) and act through the same mode of action as the parent. In addition, probes showed a slightly (nearly 3 folds) lower cytotoxicity against HeLa cells than **5** (CC_50_ (**5**) = 7.0 µM; CC_50_ (**6**) = 21.3 µM; CC_50_ (**7**) = 20.9 µM). Since the probes showed a more similar phenotypic effect on the procyclic form of *T. b. brucei* to the parent, PAL investigations were performed on such forms of the parasite [152].

In PAL experiments, probe **6** was incubated with live procyclic *T. brucei* cells, allowing target engagement first, and target capturing, upon UV photoactivation. Then, cells were permeabilized and treated with cyanine dye Cy5.5 azide for protein–probe tagging via in situ CuAAC. Finally, the labeled adducts were detected by SDS-PAGE coupled with fluorescence imaging. From protein analysis, 18 protein bands were detected to varying degrees, with the most prominent being around 70 and 55 kDa. Moreover, probe-based adducts were visualized by fluorescence microscopy, indicating that engaged targets localized in mitochondrial compartments [152].

To identify the tagged proteins, PAL experiments were coupled with LC-MS/MS analysis. Indeed, after incubation and photoactivation, the probe-labeled proteins were subjected to CuAAC with biotin azide, and the biotinylated adducts underwent pull-down experiments and enrichment with streptavidin–agarose, followed by on-bead digestion with trypsin and LC-MS/MS analysis. From this study, six mitochondrial proteins were identified as top hits, with, notably, two of them showing masses (72 kDa, 56 kDa) matching those of the most intense protein bands labeled in SDS-PAGE experiments. In particular, the two top-scoring hits matched with the most intense labeled band in SDS-PAGE experiments (~70 kDa, ~55 kDa), corresponding to the mitochondrial heat shock protein 70 (mHSP70) and ATP synthase F1 β-subunit, respectively. However, despite the prominent achieved result, no further studies have been reported so far to investigate mHSP70 as a target for B-THP-T [152].

On the other hand, a series of biochemical phenotypic studies were performed to validate or exclude the remaining five hits as putative targets for the parent compound. Since four of them corresponded to proteins involved in ATP production via proline metabolism, experiments were performed to evaluate the impact of compound **5** on cellular ATP levels. Interestingly, the compound proved to decrease ATP production in a dose-dependent way by blocking oxidative phosphorylation via FoF1-ATP synthase (mitochondrial complex V) inhibition. Moreover, PAL experiments performed with **6** in stably transfected cells expressing GFP-PTP tagged F1 α- or β-subunits confirmed that both α- and β-subunits are targeted by the probe, corroborating data from pull-down and LC-MS/MS experiments. To determine binding sites of B-THP-T within F1, docking studies were performed. Since the X-ray structure of *T. brucei* F1 was solved only in 2018 [153], and thus was not available at the time of the publication, B-THP-T was docked into F1 crystal structures from *Sacharomyces cerevisiae* (PDB entry 2WPD) and *Bos taurus* (PDB entry 1BMF). Docking of **5** within the ATP-binding sites of the α- and β-subunits indicated similar interactions, suggesting that B-THP-T compounds could act by mimicking ADP [152]. Interestingly, although the α- and β-subunits of *T. brucei* F1 share 47% and 67% sequence identity, respectively, with their mammalian homologues, they display structural differences, which could be exploited to facilitate future structure-based drug design.

Moreover, although complex V proved to be essential for both procyclic and bloodstream forms of *T. b. brucei* [154,155,156], a different role is played. Indeed, while FoF1-ATP synthase is involved in oxidative phosphorylation in both procyclic *T. b. brucei* and mammals, such a complex is mainly engaged in generating mitochondrial membrane polarization to hydrolyze ATP rather than producing it [155,157,158]. Functional differences between the two forms of *T. b. brucei* could be exploited to develop selective inhibitors of complex V of *T. b. brucei* without affecting the human host. Since most of the experiments described in such work were done on procyclic *T. b. brucei*, performing similar investigations but using the bloodstream form of the parasite could be of high interest for testing such a hypothesis.

**Figure 8:**
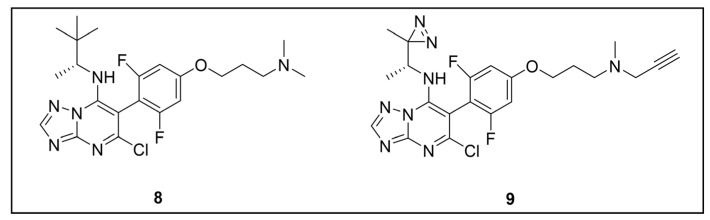
Chemical structures of compound **8** and probe **9**.

Recently, a set of microtubule targeting [1,2,4]triazolo [1,5-a]pyrimidine derivatives (TPDs) previously described for their anticancer properties [159,160,161] have displayed also antitrypanosomal efficacy [162,163]. In particular, “class II” and “hybrid” TDPs have been recently shown to be potent anti-*T. b. brucei* agents, highlighting EC_50_ values within the nanomolar range; in addition, despite showing low selectivity in vitro, administration of two selected TDPs at or below the maximum tolerated dose importantly decreased parasitemia and promoted survival in mice models [163]. However, since the efficacy of such compounds has been assessed in whole-cell assays, no information on the TPDs mechanism of action in *T. b. brucei* was provided. In order to investigate the putative target(s) of the most promising TPD, compound **8** (Figure 8), in *T. b. brucei* and provide direct insights of tubulin engagement also in the parasite, a PAL study was performed [164]. Taking advantage of SAR indications [165], probe **9** was designed and synthesized by inserting a methyldiazirine moiety suitable for cross-linking in place of the tert-butyl group present along the short alkyl chain attached to the 7-amino substituent of the parent (Figure 8). Moreover, an alkyne click handle was appended to the terminal amino group to attach a reporter tag via in situ CuAAC. To assess the effect of probe **9** on microtubule stabilization in HEK293, a microtubule-stabilization assay was performed, highlighting that the probe elicited similar effects on tubulin as the parent. Moreover, despite resulting 6–7 folds less active against both HEK293 and *T. b. brucei* (compound **8**: EC_50_ vs. HEK293 = 10.8 nM, EC_50_ vs. *T. b. brucei* = 38.9 nM; probe **9**: EC_50_ vs. HEK293 = 64.6 nM, EC_50_ vs. *T. b. brucei* = 265.3 nM), the probe resulted in being still active within the sub-micromolar range, resulting in being suited to be employed in PAL experiments. Initially, the probe was incubated with live HEK293 cells.

After photoactivation, cells were lysed, and the resulting adducts were labeled by CuAAC using a TAMRA-biotin azide to enhance both purification and detection of tagged proteins thanks to the concomitant insertion of both affinity (biotin) and fluorescence reporter (TAMRA) tags. Protein analysis highlighted a more prominent fluorescent band at ~50 kDa, which notably matched with the molecular weight of tubulin; moreover, via this experiment, two less intense bands at ~30 and ~70 kDa were also detected. Similarly, in PAL experiments performed in live *T. b. brucei* cells, a prominent fluorescent band at ~50 kDa was detected together with a less intense protein band at ~40 kDa. Interestingly, the intensity of the more intense band at ~50 kDa, detected in both HEK293 and *T. brucei* experiments, decreased in the competition assay, indicating specific labeling. Moreover, the bands at ~30 and ~70 kDa, highlighted in HEK293 only, were also affected by pre-incubation with the parent compound. Conversely, the intensity of the protein band at ~40 kDa emerging in *T. b. brucei* experiments was not affected in the competition assay, indicating that such a band does not refer to target(s) of the parent and could result from non-specific labeling. To identify the labeled proteins, proteomic investigation via LC-MS/MS analyses were performed. Indeed, fluorescent SDS-PAGE bands were excised, digested, and subjected to proteomics analysis. As expected, the ~50 kDa protein band detected in both HEK293 and *T. b. brucei* experiments was confirmed to be abundant in tubulin, indicating that such protein is the main target for the parent also in *T. b. brucei*. However, while the tubulin labeled in HEK293 consisted of different isoforms of β-tubulin, results obtained in *T. b. brucei* indicated nearly identical labeling of both α- and β-tubulin. On the other hand, proteomic analysis of the other bands detected in HEK293 showed that the ~30 kDa band contained the 40S and 60S ribosomal proteins and the high mobility group protein B1; in addition, the ~70 kDa band comprised members of the 70 kDa heat shock protein (HSP70) family of molecular chaperones (including the 71 kDa heat shock cognate and the 70 kDa heat shock proteins) and the RNA-binding protein, nucleolin. However, despite competing with the parent for binding in HEK293 experiments, these proteins were not detected in *T. b. brucei* experiments, suggesting that these proteins might be off targets for the parent, or, alternatively, additional targets occurring in HEK293 [164].

Despite the high sequence identity between mammalian and trypanosomal tubulin, reported data could indicate structural differences of TPD binding site(s) in trypanosomal and mammalian proteins. Further investigations could be of high interest since they could pave the way for the development of more potent and selective microtubule targeting inhibitors as antiprotozoal agents.

Owing to the central role played by bloodstream *T. brucei* in infection, PAL studies on such form of the parasite aim to reflect the pathological conditions and are of great interest. However, high complexity of bloodstream *T. brucei* and intrinsic limitations of PAL could contribute to yield misleading and/or false positive results that, overall, can complicate data interpretation. The membrane of bloodstream *T. brucei* is covered by a dense coat of glycoproteins (variant surface glycoprotein, VSG) known to undergo antigenic variation. Besides playing a key role in protecting the parasite from the host immune response [62,123], this complex VSG coat could importantly contribute to yield artifacts during PAL experiments. Indeed, such highly dense and complex structure could hinder the probe intracellular uptake and consequently determine non-specific labeling of such abundant glycoprotein components rather than the intended targets. In addition, by forcing the probe to localize extracellularly, the hindering effect of VSG could result in a more likely non-specific labeling of buffer components, with a consequent decrease of photo-cross-linking yield. Besides non-specific labeling of the probe, artifacts could also be ascribed to the high complexity of *T. brucei* proteome and to non-specific behavior of proteins. For instance, highly abundant and reactive proteins prone to non-specific binding could remain strongly attached to the affinity bead and are not easily removed during washing steps [34,60]. In this case, the sample obtained after chemical pull-down could be enriched with such proteins, even though the probe did not engage them. This event can greatly complicate data interpretation and analysis by increasing the complexity of the final sample. When performing PAL experiments in *T. brucei*, it is critical to be aware of such possible issues, which can be mitigated by optimizing the experimental protocol and performing additional experiments involving the parent compound (i.e., competition assay) or negative controls.

### 4.2. PAL Studies on Broad-Spectrum Anti-KDs Agents

**Figure 9:**
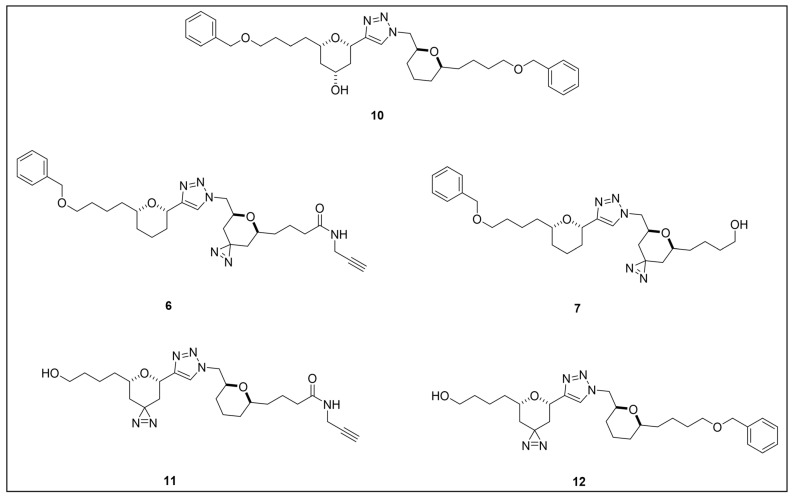
Chemical structures of compound **10** and probes **6**, **7**, **11**, and **12**.

In a follow-up study focused on acetogenins, more extensive modifications of the bis-THP scaffold were performed in order to elucidate SARs and expand the number of strategic attachment diversification points on the lead scaffold [166]. In particular, a set of *bis*-benzyl derivatives endowed with additional groups (primary alcohol and related alkyl ethers) on either THP systems were designed, synthesized, and biologically tested against *T. b. brucei* (procyclic and bloodstream forms), *T. cruzi* epimastigote, and *L. major* promastigote (a causative agent of cutaneous leishmaniasis). Although some compounds proved to elicit a certain degree of cytotoxicity on HeLa and Vero cells, as the parent compound **10** (Figure 9), some of them displayed encouraging activities against selected parasites.

Interestingly, the length of the alkyl ether substituent had a prominent impact on antiprotozoal activity. Indeed, despite being tolerated in the *T. brucei* bloodstream form, insertion of longer and bulkier groups on either THP moieties proved to reduce efficacy against other parasites. Such data indicated that THP moieties are tolerant to finely tuned modifications, and antiparasitic activity is importantly affected by sterically demanding modifications. Based on these, a set of compounds was selected and tested against other clinically relevant forms of parasites (*L. donovani* intracellular amastigote, *T. cruzi* amastigote) and on THP-1 for their cytotoxicity. Overall, selected compounds displayed higher efficacy against *T. brucei* and moderate potency against *L. donovani*, showing generally lower cytotoxicity against THP-1. On the other hand, derivatives resulted in being less promising toward *T. cruzi* amastigote, indicating that alkyl ether substituents have a higher detrimental effect in such form of parasite than in *T. cruzi* epimastigote. To elucidate the protein target(s) of this class of compounds, a set of photoaffinity labeling probes were designed and synthesized. Taking into accounts SAR findings, and, in particular, the impact of steric hindrance on antiprotozoal activity, a low-sterically demanding diazirine group was placed alternatively on either THP rings in probes **6** and **11** (Figure 9) [152]. In addition, either terminal benzyloxy moieties were replaced by propargyl amide groups, in order to provide a click handle suitable for ligation with a reporter tag. Furthermore, diazirine derivatives (probes **7** and **12**, Figure 9) [152] lacking the alkyne click handles were designed and synthesized as control compounds to probe specific labeling of photoaffinity probes. Interestingly, despite being slightly less active than methyl ether analog, probes resulted in being still active within the micromolar range, indicating that these modifications did not dramatically affect the antiprotozoal efficacy towards *T. brucei* (procyclic and bloodstream forms) and *L. major* promastigote. Differently, in agreement with SAR indications, such modifications resulted in being more impactful in *T. cruzi*, confirming that such a pathogen is more susceptible to such chemical manipulation. To test the ability of probes for selective binding and to visualize cellular localization in *T. brucei* and *L. major*, probes **6** and **11** were incubated within parasitic cells, photoactivated, and tagged with fluorescence tag Cy5.5 for fluorescence microscopy visualization. The probe proved to localize at mitochondrial compartment in both *T. brucei* and *L. major* experiments, indicating an overlapping target(s) in both parasites. Since extensive studies have demonstrated F1α/F1β subunits of ATPase as the target of such a class of compounds in *T. brucei* [152], it is likely that such protein could be the main target also in *L. major* [166]. However, more extensive studies are needed to validate such a hypothesis, as well as to validate complex V as a common target in bloodstream *T. brucei* [166].

Additionally, it could be interesting to explore the same hypothesis also in *T. cruzi* amastigote. Indeed, the lower activity of more hindered analogs against such a parasite could result from the engagement of the same target as in *T. brucei* (and *L. major*) but endowed with more stringent structural requirements. If so, important insights into the structural features of *T. cruzi* protein and structural differences with the same target of other kinetoplastids could be described. A different possibility is that the compound engages a completely different target.

Hsp90 plays a key role in protein homeostasis in cells, and its inhibition has been reported to affect a large array of client proteins [167]. Although Hsp90 has been considered extensively as a target for anticancer agents [168], the role of such protein has been also evaluated in the context of parasitic diseases [169,170,171]. In particular, Hsp90 has been found to be critical for stress adaption and survival in *Leishmania* sp. [170,172,173], but its biological function and effect on parasite proteome was unknown.

**Figure 10:**
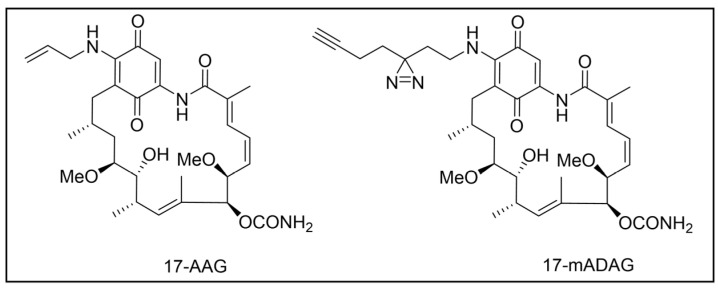
Chemical structures of 17-AAG and 17-mADAG.

To perform such investigation, the known Hsp90 inhibitor tanespymicin (17-AAG, Figure 10), which demonstrated also prominent parasite growth inhibition both in vitro and in vivo, was used as a tool to systematically investigate the impact of Hsp90 inhibition on protein synthesis in *L. mexicana* [174]. Using a combination of a mass spectrometry-based quantitative proteomics, and chemical and metabolic labeling, the authors notably provided the first protein-level evidence that Hsp90 inhibition affects global protein synthesis in *Leishmania*. Indeed, concentration- and treatment-dependent changes in the expression of many chaperones and virulence factors were detected. In particular, many ribosomal proteins proved to be down-regulated upon treatment with 17-AAG, indicating that Hsp90 inhibition importantly affects the protein synthesis capacity. Interestingly, they found that the parasite responds to chemical inhibition of Hsp90 (known as Hsp83-1 in *L. Mexicana*) by selectively down-regulating its ribosomal protein synthesis but also by increasing the production of several virulence factors and chaperones. To validate 17-AAG target engagement in live *Leishmania* parasite, a photoaffinity probe (17-mADAG, Figure 10) was designed and synthesized by including a minimalist diazirine-alkyne linker in place of the propenyl moiety of the parent. In an antiproliferative assay, 17-mADAG resulted in being slightly less active (nearly 3 folds) than the parent (IC_50_ (17-AAG) = 211 nM; IC_50_ (17-mADAG) = 640 nM), but still active within the nanomolar range and thus suitable to undergo PAL studies. The probe was used in competitive photoaffinity-based protein profiling studies combined with iTRAQ duplex labeling-based quantitative proteomic MS in *L. Mexicana* promastigotes. Briefly, promastigotes were treated with either 17-mADAG only or a combination of parent 17-AAG and the probe and then subjected to UV irradiation. Following cell lysis, extracts were clicked with biotin azide, and the labeled proteome was enriched on NeutrAvidin-agarose resin and subjected to on-bead tryptic digestion, iTRAQ duplex labeling, and LC-MS/MS analysis. As expected, Hsp83-1 was highlighted as the main target for the compound; moreover, additional 15 protein targets were detected by LC-MS/MS studies. This result further indicates that chemical Hsp90 inhibition targets multiple proteins involved in protein synthesis and quality control in the *Leishmania* parasite. However, since Hsp90 directly associates with many client proteins to form multiprotein complexes, it could be possible that, beside Hsp83-1, the additional identified proteins could result from physical association with the main target Hsp90 rather than be actual targets for 17-AAG. Despite this, the data provided in this study are very insightful since, besides confirming Hsp90 as an on-target of 17-AAG, it highlights a set of up-regulated virulence factors (i.e., superoxide dismutase, SOD), whose modulation could be promising for developing innovative drug combination options [174].

**Figure 11:**
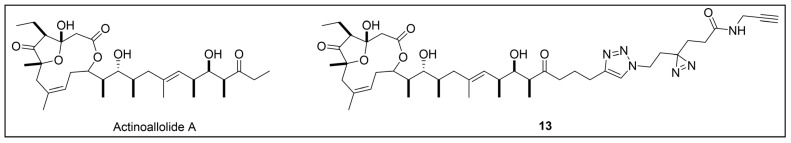
Chemical structures of actinoallolide A and probe **13**.

The actinoallolides are a family of natural polyketides, which have been isolated from the cultured actinomycete bacterium *Actinoallomurus fulvus* strain MK10-036 obtained from the roots of *Capsicum fruitescens* collected in Thailand [175]. Actinoallolides A-E were tested in vitro against *T. brucei brucei*, showing prominent antitrypanosomal activities within the micromolar–nanomolar range. Among them, actinoallolide A (Figure 11) proved to be the most potent antitrypanosomal agent, with an IC_50_ of 8.3 nM without eliciting toxicity on MRC-5 cells (CC_50_ > 100, SI > 20408), and proving to be over 5-fold more selective than the antitrypanosomal drug pentamidine. In addition, the compound showed also efficacy against *T. brucei rhodesiense* and *T. cruzi* parasites, showing an IC_50_ within the sub-micromolar range, resulting in being nearly 2-fold more potent than benznidazole. Moreover, no activity was detected against both Gram-positive and Gram-negative bacteria and against fungi and yeast, suggesting a specific antiparasitic mechanism for such a derivative. Recently, the same group set up an elegant total synthesis of the actinoallolides [176]; additionally, to explore the biological target(s) and mechanism of action of actinoallolide A, a photoaffinity probe **13** (Figure 11) was designed and synthesized. In particular, in order to produce minimal perturbation of the parent compound structure, a minimalist diazirine–alkyne moiety was appended to C_23_ of actinoallolide A. Although PAL experiments employing such a probe have not been reported yet, results from this study would be of great interest for antiparasitic drug research. Indeed, owing to the prominent and peculiar biological profile of actinoallolide A, identification of its biological target(s) and elucidation of its mechanism of action could indeed allow the generation of simplified and even more potent analogs as potential innovative and effective antiprotozoal agents [176].

## 5. Malaria and Therapeutic Options

Malaria is a devastating disease, which caused 608,000 deaths in 2022 worldwide, as reported by WHO in the world malaria report in 2023. From 2021 to 2022, the global number of malaria cases increased 5 million to reach a total number of 249 million cases. The reported additional cases observed between 2021 and 2022 mainly occurred in five countries: Pakistan, Ethiopia, Nigeria, Uganda, and Papua Nuova Guinea [7].

**Figure 12:**
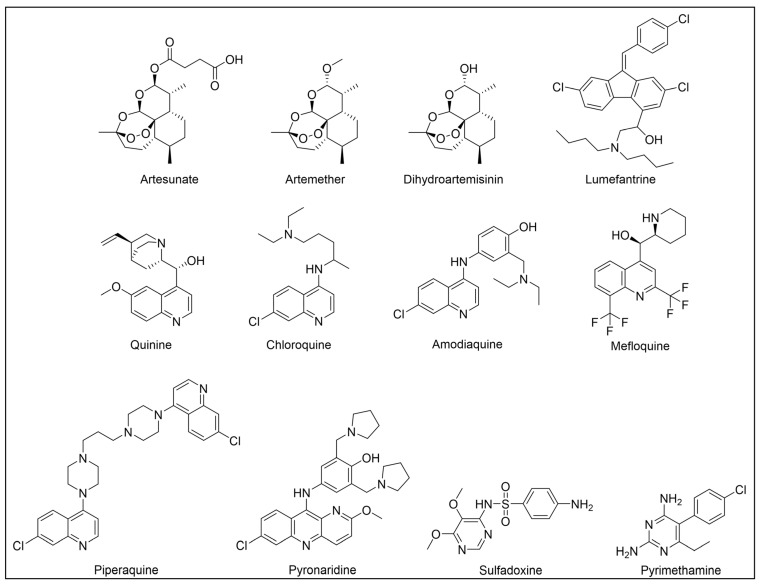
Structure of drugs employed in the treatment of Malaria.

Malaria is transmitted by female *Anopheles* mosquitoes and is caused by protozoans from the genus *Plasmodium*, being *Plasmodium falciparum*, and *P. vivax*, the most important species causing infections. Severe malaria is usually treated by intravenous artesunate as first-line treatment. Second-line treatments consist of intramuscular artemether if artesunate is not available, or intravenous quinine only if parenteral artemisinin-based therapies are not available. A combination therapy consisting of quinine and artesunate is recommended for severe malaria in those regions with artemisinin resistance. On the other hand, uncomplicated malaria caused by *P. falciparum*, *P. knowlesi*, and chloroquine-resistant *P. vivax* is treated with approved artemisinin-based combination therapies (i.e., artemether–lumefantrine, artesunate–mefloquine, dihydroartemisinin–piperaquine, artesunate–amodiaquine, artesunate–sulfadoxine–pyrimethamine, artesunate–pyronaridine). Differently, infections caused by *P. malariae*, *P. ovale*, and non-resistant *P. vivax* strains are treated with chloroquine (Figure 12) [177].

Many prevention strategies have been introduced during the years comprising chemoprophylaxis, mosquito vector control strategies such as insecticide-treated mosquito nets and indoor residual spraying, and vaccination [7]. Since 2021, a first malaria vaccine (i.e., RTS, S) was recommended by WHO to prevent malaria among children living in regions with high-rate infections caused by *P. falciparum*. A malaria vaccine implementation program coordinated by WHO in Ghana, Kenya, and Malawi allowed the treatment of 2 million children with one dose minimum and resulted in a 13% reduction of early childhood deaths. At the end of 2023, WHO recommended a second safe and effective vaccine, namely, R21, that will make it possible to reach a greater number of children living in regions with a high risk of malaria [7,177]. Progress toward elimination of malaria are evident in many countries, in accordance with the 2023 world malaria report. On the other hand, some countries are experiencing a resurgence of malaria and will need extensive efforts in the coming years to reverse the trend [7].

Resistance to available therapies is also a growing concern that drug hunters are trying to avoid by developing new candidate drugs. A recent review described the antimalarial development pipeline and focused on the approaches currently followed for the discovery of new antimalarial drugs [178]. Target-based screening is used in antimalarial drug discovery to identify molecules that possesses high potency and selectivity against a defined target. However, these lead compounds identified with target-based screening usually need to be optimized to guarantee antiplasmodial activity in whole-cell cultures [178]. Many targets have been identified for antimalarial drug discovery in different campaigns, and some of them were validated in clinical studies [178], for example, cytochrome B [179], heme [180], dihydrofolate reductase–thymidylate synthase [181], dihydropteroate synthase [182], deoxy-D-xylulose-5-phosphate reductoisomerase [183], prolyl tRNA synthetase [184], 70S ribosome [185], dihydroorotate dehydrogenase [186,187,188], *Pf*ATPase4 [189], phosphatidylinositol 4-kinase [190], elongation factor 2 [191], and multidrug resistance protein 1 [192].

On the other hand, phenotypic screening, as previously examined in detail, is another important technique employed to identify new antimalarial drugs with potentially new mechanisms of action [178]. In this scenario, the putative mechanism of action of a new drug candidate developed by the phenotypic screening approach needs to be discovered. On the other hand, the mechanism of action of some old drugs such as artemisinin and chloroquine were not completely understood and need to be better investigated. To meet the need, researchers around the world are working and are trying to take advantage of the PAL technique to uncover the mechanisms of action of these drugs. In the following section, PAL approaches used to identify the mechanism of action of several approved or candidate antimalarials are described.

## 6. PAL Studies on Malaria

### 6.1. Artemisinin-Based Probes

**Figure 13:**
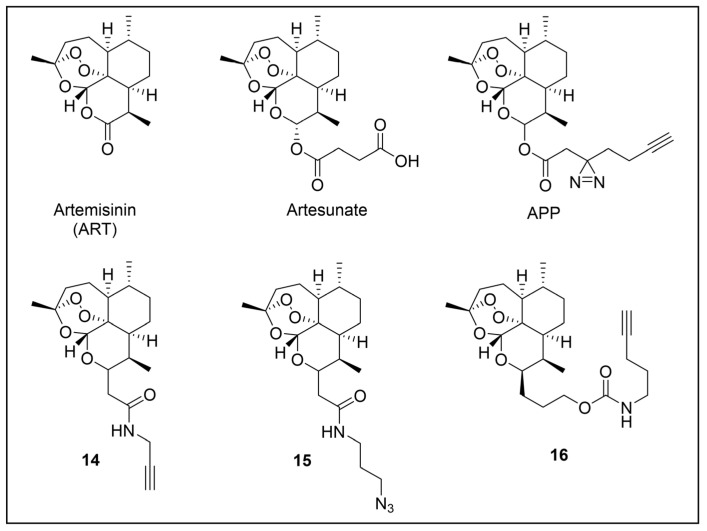
Structures of Artemisinin, Artesunate, probes **14**–**16**, and photoaffinity probe APP.

The mechanism of action of artemisinin (ART) has been largely investigated in the past, proving that the activation of the endoperoxide bridge mediated by ferrous heme and consequent alkylation of parasite proteins and lipids is leading to plasmodium death [193,194]. ART-heme adducts were also proved to be an inhibitor of β-hematin crystallization and heme detoxification [195]. Several proteins essential for parasite survival involved in glycolytic, protein synthesis, hemoglobin degradation, and antioxidant defense have also been identified as targets of ART. These latter results were obtained using activity-based protein profiling (ABPP) that took advantage of the ART probe containing click chemistry handles (alkyne or azide compounds **14**–**16**, Figure 13) to search the covalent proteins target of ART [196].

On the other hand, to uncover the non-covalent targets of ART, photoaffinity labeling has been reported for the first time in 2023 [197]. In this article, an ART photoaffinity probe (APP, Figure 13) was designed and synthesized (please refer to Table S2 for IC_50_ data). The pharmacophoric moiety of ART containing the endoperoxide bridge was conjugated with minimalist diazirine as the photoreactive moiety and an alkyne click-chemistry handle for chemical proteomic studies. The specificity of this chemical probe was further confirmed by competition experiments [197,198]. Fluorescence labeling experiments were conducted on lysate of infected red blood cells (iRBCs) incubated with probe, with and without UV irradiation, followed by reaction with TAMRA azide, SDS page separation, and fluorescence scanning for visual analysis. This experiment showed that ART binds to proteins with non-covalent binding in addition to covalent binding, demonstrated by a slightly increased fluorescence of the UV irradiation group compared to the non-UV irradiation group. Chemical proteomics analysis, conducted on lysate of iRBC incubated with probe, with and without UV irradiation, followed by reaction with biotin azide, pulled down on streptavidin beads and on-beads digestion, TMT labeling, and LC-MS/MS analysis, identified 77 protein targets in the UV irradiation group and 41 protein targets in the non-UV irradiation group, with 32 overlapping proteins in the two groups.

Protein–protein interaction analysis revealed that interaction networks focused on GTP hydrolysis, histone deacetylases, and protein folding. Moreover, gene ontology studies showed that the pathways of glucose production and glucose metabolism were enriched. In particular, triosephosphate isomerase (*Pf*TIM) seems to be a non-covalent target of ART, L-lactate dehydrogenase (*Pf*LDH) is likely a covalent target, and glygeraldehyde-3-phosphate dehydrogenase (*Pf*GAPDH) seems both a covalent and non-covalent target of ART [197].

The same research group provided an advancement using the same photoaffinity probe (i.e., APP) to evaluate the protein target of ART in different stages of the intraerythrocytic developmental cycle (IDC) of *P. falciparum*, that is to say, the ring, trophozoite, and schizont stages [198]. These experiments were conducted on living parasites at different developmental stages, followed by analysis of soluble proteins upon extraction. Fluorescent labeling, pull-down assay, and bioinformatic analysis showed that the targets of ART are involved in protein catabolic processes, proteolysis at the ring stage and trophozoite stage. On the other hand, targets are involved in peptide metabolism, amide biosynthesis, and translation at the schizont stage. Moreover, the authors supplied results proving that ART exerts antimalarial activity by blocking protein synthesis, interfering with the glycolytic energy supply pathway and disrupting redox-related processes [198]. The latter article provides an invaluable advancement toward the discovery of a non-covalent target of ART in living parasite cells at different developmental stages. However, considering that *Pf*LDH and *Pf*GADPH are both involved in glucose metabolism and seem to be targets of ART and related probes, a further study using an ART probe to evaluate possible intraerythrocytic differences of such enzyme expression and function in the different developmental stages would be intriguing.

### 6.2. Chloroquine-Based Probes

Chloroquine is a well-known antimalarial drug extensively used in the past and still used nowadays for the treatment of *P. vivax* sensitive strains [177]. It is a weak base that, once entering and being protonated, remains trapped in the acidic digestive vacuole. In this compartment, chloroquine inhibits hematin detoxification by inhibiting the formation and growth of hemozoin crystals. Therefore, accumulation of toxic hematin results in digestive vacuole membrane puncture leading to *Plasmodium* death. Moreover, chloroquine inserts into the DNA helix, leading to a DNA-chloroquine complex that inhibits *Plasmodium* growth and reproduction [199].

Despite this accepted mechanism of action, to widen the knowledge of further molecular mechanisms that could mediate the antimalarial activity of chloroquine, some research groups have developed and synthesized photoaffinity probe analogues of chloroquine during the years.

**Figure 14:**
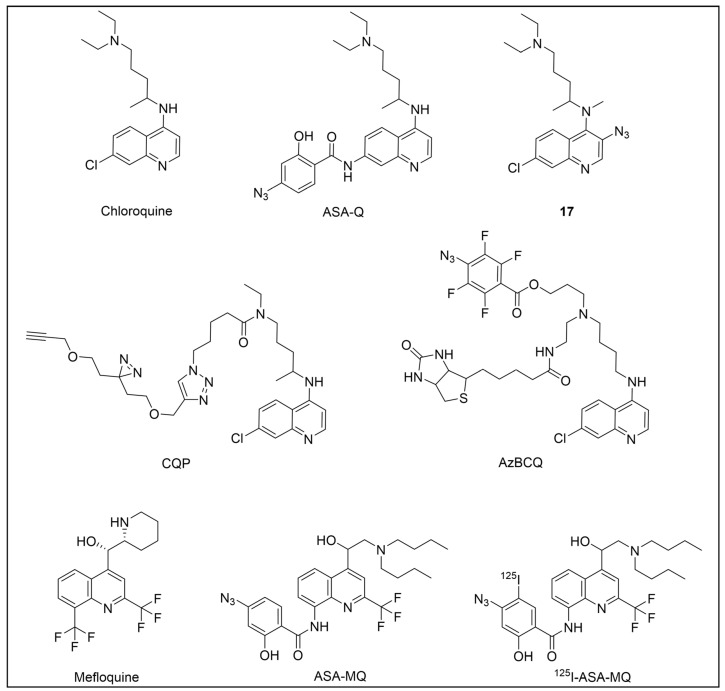
Structures of chloroquine, mefloquine, and photoaffinity probes.

The first chloroquine probe, namely, *N*-(l-(l-diethylamino-l-methylbutylamino)quinolin-6-yl)-4-azido-2-hydroxybenzamide *(*i.e., ASA-Q, Figure 14), was synthesized and published in 1994 by Foley et al. [200]. The activity of ASA-Q against a chloroquine-sensitive *P. falciparum* was only partially reduced compared to chloroquine, despite the molecule not including a chloro-substituent of the parent molecule; indeed, the growth of this *P. falciparum* clone (3D7) was inhibited by ASA-Q with an IC_50_ of 18 ng/mL compared with an IC_50_ of 11 ng/mL of Chloroquine. The probe was designed bearing an aromatic azide that allows binding with proteins via nitrene intermediates upon UV light exposure. Radio-iodinated ASA-Q was therefore employed in photolabeling experiments using malaria-infected erythrocytes that allowed identifying two chloroquine-binding proteins with apparent molecular masses of 42 and 33 kDa via SDS-polyacrylamide gel electrophoresis followed by visualization using phosphorimage analysis. The specificity of binding to these proteins was also ensured by competitive experiments with chloroquine that prevented iodo-ASA-Q binding [200].

In a follow-up study [201], the same authors identified the 33 kDa protein from *Plasmodium falciparum* that was previously detected via photoaffinity labeling studies, being the *P. falciparum* lactate dehydrogenase (*Pf*LDH). The protein was identified upon purification via *N*-terminal amino acid sequence analysis and further confirmed by immunoprecipitation using anti-*Pf*LDH antibodies. However, the activity of the purified enzyme was not significantly inhibited by chloroquine, suggesting that the enzyme is not a direct target of the molecule. On the other hand, *Pf*LDH was inhibited by the free heme (i.e., ferriprotoporphyrin IX (FP), IC_50_ of 0.2 μM). The author also proved that chloroquine protects *Pf*LDH from FP inhibition, suggesting that chloroquine can interact with *Pf*LDH and affect the binding of other molecules [201].

More recently, a novel probe named CQP (Figure 14), able to combine the photoaffinity labeling and click-chemistry functionalization approaches, was designed and synthesized (Figure 14) [202]. The structure of CQP maintains the quinoline scaffold while including a minimalist diazirine moiety via an acyl triazolyl linker. The probe retained the nanomolar antimalarial activity of chloroquine and was used in an affinity-based protein profiling study identifying 40 protein targets. At the same time, probe-free mass spectrometry-coupled cellular thermal shift assay (MS-CESTA) identified 83 putative protein targets. The two methods commonly identified eight protein targets that were all involved in glycolysis and energy metabolism. Detailed experiments conducted on a selection of these targets, namely, *Pf*LDH, *P. falciparum* ornithine aminotransferase (*Pf*OAT), *P. falciparum* pyruvate kinase (*Pf*PyrK), *P. falciparum* phosphoglycerate kinase (*Pf*PGK), and *P. falciparum* triosephosphate isomerase (*Pf*TPI), demonstrated functional binding of CQP to the target enzymes, proving that chloroquine exerts antimalarial activity modulating protein involved in glycolysis and energy metabolism [202]. It is worth noting that CQP identified only 40 protein targets in PAL experiments compared to 77 proteins identified by the ART probe APP. Despite considering that these are different drugs with diverse putative protein targets, the difference might be due to the different nature of the target engagement made by the drug (i.e., non-covalent versus covalent).

The PAL approach was also used to study the resistance mechanism to chloroquine. In particular, some theories propose *P. falciparum* chloroquine resistance transporter (*Pf*CRT) as being responsible for active transport, or facilitative diffusion, of chloroquine from the digestive vacuole to the cytosol. To better study the molecular interactions between the molecule and purified *Pf*CRT protein, a chloroquine analogue was designed and synthesized as a photoaffinity probe (i.e., AzBCQ, Figure 14) [203]. AzBCQ contains a perfluorophenylazido moiety for photoaffinity labeling and a convenient biotin tag. The specificity of the probe was ensured by competition experiments with chloroquine. The study allowed the authors to determine that the probe had a unique covalent attachment site in the digestive–vacuolar-disposed loop between putative helices 9 and 10 of *Pf*CRT, and to propose that the binding site is formed by helices 1, 9, and 10 [203]. The perfluorophenylazido moiety used in this example for photoaffinity labeling is a convenient analytical handle useful for proteomic studies.

A new photolabile probe containing an aryl azide photoreactive moiety in the 3-position of quinoline and minimal modification to the structure of chloroquine has been recently synthesized and characterized (probe **17**, Figure 14) [204]. The authors reported the synthesis, photoaffinity properties, and nitrene insertion experiments. Photo irradiation at 365 nm did not induce decomposition of the aromatic system, as observed at 254 nm, and allowed obtaining a new compound via loss of nitrogen. Photolysis studies allowed detecting the insertion products only in experiments with tetracyanoethylene, while adduct products with GlyGlyGly and *N*-phenylmaleimide were not detected [204].

Altogether, these examples provide advancements in the search for multiple mechanisms of action of chloroquine derivatives employing structurally diverse probes, which were designed following different strategies.

### 6.3. Mefloquine-Based Probes

Mefloquine is an efficacious antimalarial agent expressing its activity through different mechanisms of action. It has been proved that mefloquine interacts with the cytosolic 80S ribosome of *Plasmodium falciparum*, inhibiting protein synthesis [205]. Mefloquine also binds to *Plasmodium falciparum* acyl-CoA binding proteins, inducing loss of function and degradation, thus inhibiting *Plasmodium* growth and proliferation [206]. Moreover, mefloquine showed inhibition of heme polymerization and reduction of proteolytic activity targeting *Plasmodium falciparum* metacaspase-1 protein [207].

In the past century, the mefloquine binding proteins were investigated with a photoreactive mefloquine analogue, namely, ASA-MQ (Figure 14). The synthesis of the probe was described, and parasite growth inhibition experiments showed only a 10-fold reduction compared to mefloquine. The radio-iodinated probe (i.e., ^125^I-ASA-MQ, Figure 14) was then used to find possible drug targets incubating the probe with erythrocytes infected with trophozoite-stage parasites of D10 strain *P. falciparum*. Both 22 kDa and 36 kDa proteins were identified as specific parasite binders. ASA-MQ also allowed identifying a serum protein, apo-A1, suggesting that this and other high-density lipoproteins (HDLs) may be responsible for the serum depot of mefloquine [208].

### 6.4. Plasmodione-Based Probes

**Figure 15:**
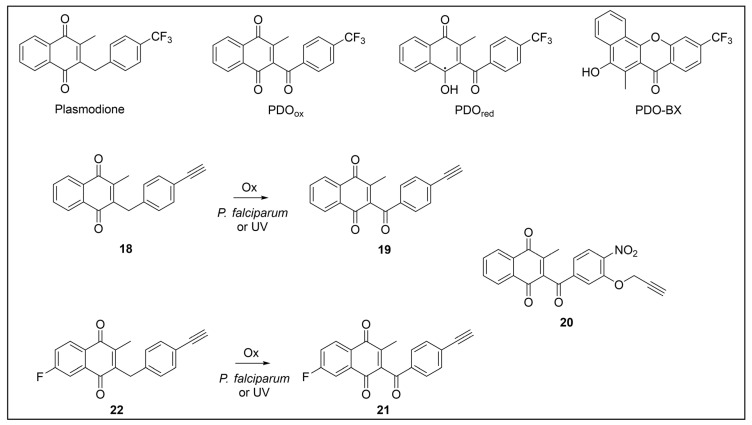
Structure of plasmodione, PDOox, PDOred, PDO-BX, and probes **18**–**22**. Ox = oxidation in vivo.

Aiming to evaluate the mechanism of action of plasmodione, an early lead compound with potent antimalarial activity, a series of (pro-)activity-based protein profiling probes was developed [209,210]. Plasmodione has been previously proposed to be metabolized into 3-benzoylmenadione (PDO_ox_), reduced 3-benzoylmenadione (PDO_red_), and a benzoxanthone metabolite (PDO-BX) (Figure 15). These metabolites contribute to the mechanism of action of plasmodione and, indeed, could redox cycle with several oxidoreductase and disturb key parasite processes, such as impeding the detoxification of free heme thus leading to parasite death [211,212].

Considering the mentioned metabolite activity, ABPP probes were designed not only starting from PD, but also starting from PDO_ox_. These new probes bear a clickable alkyne and a benzylic moiety that can be activated by in vitro benzylic oxidation to a benzoyl photoreactive group (e.g., compound **18** in Figure 15), or a clickable alkyne moiety and a benzoyl photoreactive group (e.g., compounds **19** and **20** in Figure 15) [209]. Probes **19** and **20**, with a *para*-alkyne or *para*-NO_2_ substituent, were found to be the best photoreactive compounds of the first series, with compound **19** being also the best clickable compound. Upon ensuring the cross-linking ability of probes using *N*-acetyl-methionine methyl ester and protein models such as GSH, a proof-of-concept of probe **20** application with isolated *human* and *Plasmodium falciparum* glutathione reductase (GR) was evaluated, proving the formation of probe **20**-GR adducts. Subsequent click reaction with biotin-azide and avidin pull-down of labeled GR proved the applicability of the probes. Of note, glutathione reductase has been postulated to be responsible for the formation of benzoxanthone (PDO-BX), a methabolite of 3-benzoylmenadione (PDO_ox_). The antimalarial activity of compounds **19** and **20** (1806 and 417 nM IC_50_ respectively) showed an IC_50_ of 20–90-fold higher than plasmodione (20 nM IC_50_). On the other hand, probe **18** showed an antimalarial activity comparable with plasmodione with an IC_50_ of 49 nM. This probe can be converted into probe **19** by UV-photoirradiation, as demonstrated by the authors, and is expected to be metabolized into probe **19** in living *Plasmodium* parasites [209].

In a follow-up study [210], the same research group optimized the click reaction conditions in an aqueous solution using probes **19** and **20** with tetrakis(acetonitrile)copper (I) and bathocuproinedisulfonic acid as the ligand. The photo reactivity of probe **19** was also improved designing a new probe (**21**, Figure 15) substituted in position 6 with a fluorine atom. The benzylic probe **22** (Figure 15) is also described as a precursor of **21**, being susceptible to enzymatic oxidation in living cells [210].

Considering that yeast is sensitive to PD, probe **21** was used in this model system as a proof of concept. Upon lysis of *S. cerevisiae*, incubation of probe **21** with proteins and photoirradiation at 350 nm ensured target protein–probe binding. Click reaction with biotin and purification on streptavidin beads allowed the identification of 11 target proteins statistically more abundant than in the control. Of these 11 proteins, 3 are involved in oxidoreductase activity [210].

Probe **22** was then used to evaluate the affinity-based protein profiling on *P. falciparum* parasites. Red blood cells infected with the *P. falciparum* NF54 strain were incubated with probe **22**, irradiated, and lysed. Proteins were then subjected to click chemistry and pulled down for nano LC-MS/MS analysis. The *P. falciparum* (NF54 strain) proteome was used by the authors to analyze the results. In total, 44 bound proteins were identified to be statistically different from the control. Most of the enriched proteins, comprising a glycophorin binding protein, two mitochondrial chaperonins, and profilin, are essential in the parasite life cycle. Of note, the antimalarial activity of probe **22** is lower than PD but still effective against strain NF54 of *P. falciparum*, with an IC_50_ of 179.2 nM compared to the 46.6 nM IC_50_ of plasmodione. Further studies will probably validate these targets to elucidate a detailed mechanism of action of plasmodione [210].

### 6.5. Miscellaneous Probes

**Figure 16:**
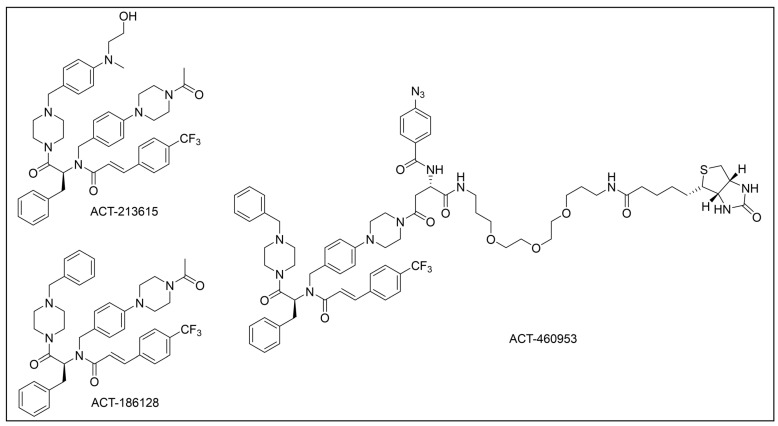
Structures of ACT-213615, ACT-186128, and ACT-460953.

A photoreactive affinity probe was developed to study the mechanism of action of a class of antimalarial compounds discovered in a cell-based anti-proliferative screen [213,214]. ACT-213615 (Figure 16), one of the most important compounds of the series, inhibited the growth of *Plasmodium falciparum* with an IC_50_ ranging from 0.4 to 2.7 nM, depending on the strain used, a result comparable with chloroquine [213]. ACT-186128 (Figure 16), an analogue of the previously mentioned ACT-213615, was used as the starting point for development of the photoreactive probe modulating the terminal phenylpiperazine moiety with B1-NHS cross-linking and the capturing tool from Caprotec Bioanalytics GmbH (Berlin, Germany).

The activity of the probe (i.e., ACT-460953, Figure 16) was compared to ACT-186128 and proved that the modified moieties did not impact compound activity. Indeed, the anti-proliferative activity against *P. falciparum* 3D7 of ACT-460953 resulted in an IC_50_ of 34.1 nM, comparable with the 14.9 nM IC_50_ of ACT-186128. The probe was used in fluorescent imaging experiments proving that the compound is a suitable capture reagent capable of entering into the cytoplasm of parasites, and then used in pull-down experiments that identified 20 potential compound targets. A functional test performed on isolated proteins allowed the authors to find physical and functional interactions between ACT-213615 and the *P. falciparum* multidrug resistance protein 1 (*Pf*MDR1). This finding suggests that *Pf*MDR1 may play a role in the antimalarial activity of this class of compounds, but the authors are still uncertain if it could be the actual molecular target of ACT-213615 [214].

**Figure 17:**
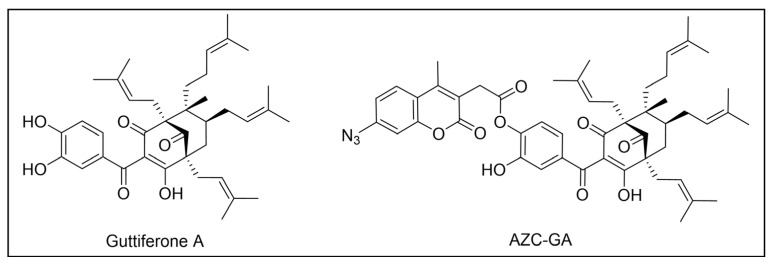
Structures of guttiferone A and AZC-GA.

Guttiferone A (GA, Figure 17) is a natural compound isolated from *Symphonia globulifera* (Clusiaceae) that possesses inhibitory activity against multiple strains of *Plasmodium falciparum*, with an IC_50_ ranging from 0.5 to 3.3 μM [215,216]. The target proteins responsible for the antimalarial activity remain elusive. To meet the need of discovering targets of the active molecule, a photo alkylative fluorogenic probe was developed, namely, AZC-GA (Figure 17) [217]. Following a preliminary medicinal chemistry study to understand the modifiable portion of the molecule, a 7-azidocumarin (AZC) photoreactive and fluorogenic moiety was introduced by modification of the catechol portion. In vitro antiplasmodial activity confirmed that probe AZC-GA maintained the inhibitory activity (5.4 μM IC_50_) possessed by guttiferone A (5.1 μM IC_50_). Fluorogenic photoactivation experiments with AZC-GA in *P. falciparum*-infected erythrocytes demonstrated specific labeling upon photo irradiation at 359 nm, which was absent in the cytosol of erythrocytes but also in non-infected erythrocytes. Co-administration of guttiferone A (20-fold excess compared to AZC-GA) induced a strong reduction of photolabeling, even though it was not completely inhibited, thus indicating non-specific binding. The authors did not report target identification using this probe and indicated that, based on the mentioned results, a second-generation probe with diminished non-specific binding should be developed [217].

**Figure 18:**
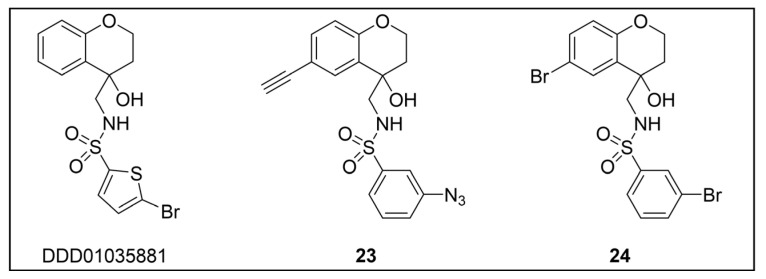
Structures of DDD01035881, probe **23**, and parent compound **24**.

A high-throughput screening (HTS) recently identified transmission blocking agents from the Global Health Chemical Diversity Library (GHCDL). DDD001035881 (Figure 18), belonging to *N*-[(4-hydroxychroman-4-yl)methyl]-sulphonamide class of compounds, represents the most potent inhibitor of the *Plasmodium* male gametes from mature stage V male gametocytes [218]. To study the mechanism of action of this new class of compounds, photoaffinity labeling and label-free cellular thermal shift assay were employed. Structure–activity relationships were used to develop a photoaffinity probe (probe **23**, Figure 18) bearing both an alkyne handle and an azide as the photoreactive group, capable of maintaining inhibitory activity in the micromolar range (IC_50_ 3981 nM) compared to nanomolar activities of the parent molecule **24** (IC_50_ 785 nM, Figure 18) and DDD001035881 (IC_50_ 292 nM) [219].

Live *Plasmodium falciparum* stage V gametocytes were treated with probe **23**, irradiated at 254 nm, and then purified and lysed upon ligation with biotin capture reagent (AzTB) via copper-catalyzed azide-alkyne cycloaddition (CuAAC). The proteins were enriched with NeutrAvidin agarose beads and prepared for 9plex tandem mass tag (TMT) labeling and quantification, followed by liquid chromatography in tandem with mass spectrometry. This approach allowed identifying a gametocyte-specific 16 kDa protein as the possible target being the *P. falciparum* PVM protein Pfs16. The target protein was also confirmed using the label-free cellular thermal shift assay. The authors demonstrated that the class of compounds inhibits microgametogenesis with activity in the first 0–6 min post activation of gametocytes and validated the series of compounds as leads for the development of new transmission-blocking antimalarials [219].

**Figure 19:**
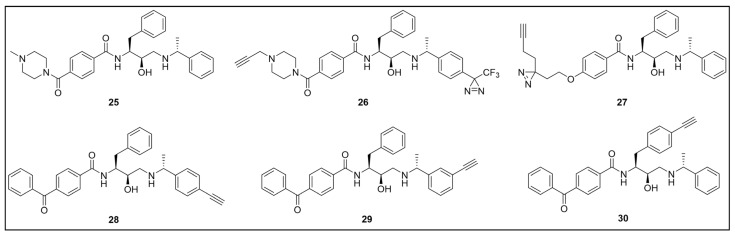
Structures of **25** and chemical probes **26**–**30**.

A series of hydroxyethylamine derivatives have been previously investigated as potential antimalarials targeting *Plasmodium falciparum* aspartic proteases (i.e., plasmexins I–X, *Pf*PMI–X). Compound **25** (Figure 19) has been characterized as a potential dual inhibitor of plasmexins IX and X, impeding parasite egress from the parasitophorous vacuolar membrane and red blood cells and preventing erythrocyte invasion [220]. Aiming to better study the mechanism of action of these compounds, new chemical probes were designed and synthesized to be used in future experiments for target protein identification. Two probes, namely, compounds **26** and **27** (Figure 19), incorporated a photoreactive diazirine moiety as the photoreactive group, while other chemical probes (e.g., compounds **28**–**30**, Figure 19) were designed with a benzophenone photoreactive moiety [221]. The probes were designed without modification of the important hydroxyethylamine moiety and with a convenient clickable alkyne handle. The antimalarial activity was then explored to understand the impact of various structural modifications. Parasite viability after 72 hours upon compound incubation was measured using *P. falciparum* chloroquine-sensitive (D10) and chloroquine-resistant (W2) strains, showing sub-micromolar activity for all probes except for **26**.

The most potent compound in this assay was **29**, with the benzophenone photoreactive group, showing an IC_50_ < 20 nM against both *P. falciparum* strains. A *Pf*PMX enzymatic assay of selected chemical probes showed that the antimalarial activity observed for **25** and chemical probes including **29** is at least partially due to inhibition of this enzyme, and the probes will serve in the future to determine the selectivity for plasmexin IX and X over other targets [221].

**Figure 20:**
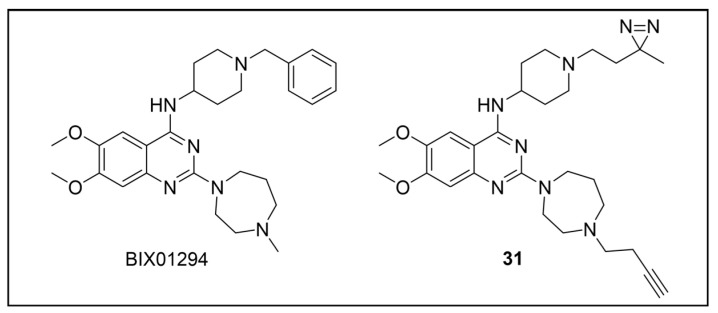
Structures of BIX01294 and probe **31**.

A series of compounds containing the diaminoquinazoline scaffold has been recently investigated as potential *Plasmodium* histone lysine methyltransferase inhibitors (*P*HKMT). BIX01294 (Figure 20), an inhibitor of *human* histone lysine methyltransferase (e.g., G9a), showed antimalarial activity (IC_50_ of 43.4 nM against *P. falciparum* 3D7 strain) [222,223]. Moreover, BIX01294 decreased levels of histone H3K4Me3 after treatment of *P. falciparum* in culture, suggesting that the compound could inhibit *Plasmodium* histone lysine methyltransferase [223]. To better understand the target protein of this class of compounds, a photoaffinity probe was designed for chemical proteomics studies. Probe **31** (Figure 20), which was developed following SAR previously discovered [222], includes a diazirine photoreactive moiety and a terminal alkyne group suitable for click chemistry reactions and proteomic experiments [224]. The probe exhibited similar antimalarial activity (IC_50_ = 42 nM) compared to BIX01294. Aiming to identify protein targets of the class of compounds, probe **31** was incubated with the lysate of blood stage *Plasmodium falciparum* (3D7 strain), irradiated with UV light and clicked with an azide-TAMRA-biotin (AzTB) for purification and enrichment of linked protein targets. Liquid chromatography in tandem with mass spectrometry identified 104 proteins from different classes as putative targets of BIX01294 and analogues. Many identified proteins are essential for *P. falciparum* and *P. bergei*, despite *P*HKMT not being identified in the list of putative targets. This finding could be due to methodology limitations or that *P*HKMT are not the major target of this class of compounds [224]. This study would benefit from a follow-up photoaffinity labeling experiment conducted in live parasites that could afford different results.

**Figure 21:**
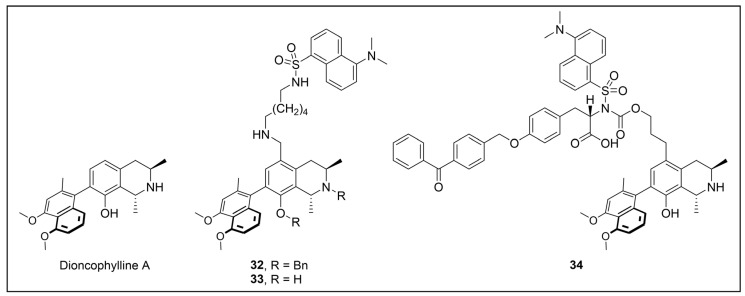
Alkaloid dioncophylline A and its probes **32**–**34**.

Naphthylisoquinoline alkaloids extracted from lianas of the tropical plants Dioncophyllaceae and Ancistrocladaceae have shown in vitro and in vivo (mouse model) antiplasmodial activities against *P. falciparum* and/or *P. bergei* [225,226,227]. To evaluate the mechanism of action of these alkaloids, fluorescent and photoreactive analogues of dioncophylline A (Figure 21) were designed and synthesized. The fluorescent dansyl moiety was chosen as the fluorophore, yielding compounds **32** and **33**, while the benzophenone group was chosen as the photoreactive group to be incorporated, in addition to the dansyl fluorophore, into probe **34** (Figure 21). The antiplasmodial activity of compound **32** (0.045 μM IC_50_) showed increased activity compared to the parent molecule dioncophylline A (0.381 μM IC_50_) and compound **33** (0.602 μM IC_50_), while the photoreactive probe **34** displayed slightly weaker antiplasmodial activity (0.96 μM IC_50_) compared to the parent molecule. Fluorescent studies with compounds **32** and **33** showed that the compounds accumulate only in infected erythrocytes. Moreover, the cytoplasm of infected erythrocytes was unlabeled, and the fluorescence was restricted only in the parasite. Microscopic studies on compound **34** agreed with previous ones, thus demonstrating that this photoreactive and fluorescent probe is suitable for photoaffinity labeling studies [228].

**Figure 22:**
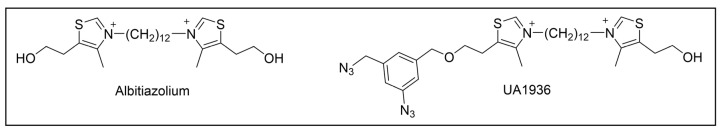
Structures of albitiazolium and probe UA1936.

Albitiazolium (SAR97276, Figure 22) is a choline analog that exhibits potent in vitro antimalarial activity against *P. falciparum* and was previously investigated as a clinical drug candidate [229,230]. This compound accumulates in the parasite and inhibits the parasite choline intake, as well as the activity of enzymes involved in the de novo synthesis of phosphatidylcholine [231]. Aiming to evaluate the mechanism of action of albitiazolium, a probe (UA1936, Figure 22) containing a phenyl azido moiety and an alkylazido group was designed to ensure photoreactivity with the target protein and click chemistry for protein isolation and purification [232]. The new probe possesses comparable in vitro growth inhibition to albitiazolium, with IC_50_ values of 4.5 nM for UA1936 and 4.2 nM for albitiazolium. Treatment of saponin-freed parasites with UA1936, followed by irradiation at 254 nm, parasite lysis, and isolation of the target protein via immobilization of probe–protein complexes with alkyne agarose resin, allowed the authors to investigate the target proteins via LC-MS/MS and cluster them on a gene ontology term using different bioinformatic packages. Eleven proteins were found interacting with UA1936. These proteins are involved in glycerophospholipid metabolism, phospholipid binding, phospholipid transport, and vesicular transport functions [231,232].

Interestingly, among the potential targets identified, choline/ethanolamine phosphotransferase, the essential enzyme responsible for the last biosynthetic step of phosphatidylcholine and phosphatidylethanolamine, was the sole significantly displaced enzyme in the presence of albitiazolium. This finding further supports the proposed mechanism of albitiazolium [231,232].

## 7. Conclusions

We are experiencing an era of renewed interest in the search for therapeutic options for malaria and KDs. In recent years, increasing efforts have been devoted to the field, and, indeed, new antiprotozoal agents have been identified, with some of them being advanced in clinical trials, and a few being approved as antiparasitic drugs. Most of these new chemical entities were discovered via phenotypic approach that was followed by TD strategies to better understand their biological targets. In this article, we have reviewed many studies that employed PAL as a TD strategy to uncover the mechanism of action of these new chemical entities or to deepen the knowledge of approved drugs. The majority of the recent applications of PAL in the antiprotozoal field have focused on malaria, but some studies were also performed to elucidate the molecular basis of new anti-kinetoplastid agents’ function.

From a medicinal chemistry point of view, the design of chemical probes is a critical step in PAL experiments. Most probes reported in this review bear a reactive diazirine moiety as the photoreactive group and an alkyne as the chemical handle. However, the employment of aromatic azides or benzophenone groups as photoreactive moieties was also reported in some reviewed research articles. Considering the available chemical handles, the alkyne moiety was highly preferred compared to alkyl azide, whereas some studies also reported the insertion of a biotin tag, fluorophores (e.g., dansyl group), or radio nuclides (i.e., ^125^I) in place of the chemical handle for target detection and identification.

In general, several probes of antiplasmodial and anti-kinetoplastid agents were conclusive at identifying drug targets or biomolecules responsible for resistance mechanisms. As an example, PAL experiments were useful for identifying possible non-covalent targets of artemisinin using live parasites at different developmental stages and for deepening knowledge on the mechanism of action and resistance mechanism of chloroquine. Of note, most reported PAL experiments were conducted using live parasites that usually afford more reliable results compared with experiments conducted using cell lysates. It was also worth noting that some recent studies not only reported studies on clinically relevant protozoan species but also conducted PAL experiments using parasites at different developmental stages, which facilitates the identification of key biological targets in different forms of pathogens.

Considering the data presented in this review, an increased employment of PAL studies in the field of anti-kinetoplastid agents would be beneficial for drug discovery purposes. Indeed, a more widespread use of PAL could aid in elucidating the complex parasite’s biology, which is an important criticism in the rational development of new antiprotozoal drugs. In turn, the application of such methodology could be useful to elucidate potential new (or previously unexplored) biological targets of parasites. Noteworthy, drug profiling of phenotypically active compounds via PAL could provide insights into the druggability of deconvoluted biological targets, paving the way to apply a target-based drug discovery strategy also in this field of research. The application of both these drug discovery strategies could be critical to render more focused, rational, and fruitful the current research efforts, and overall expedite the development of new antiprotozoal agents.

Besides allowing for understanding the molecular basis of drug efficacy, PAL can also provide important insights on compounds’ off-targets. Considering that toxicity is a critical issue for currently employed antiprotozoal drugs, gaining such information could provide indications for chemically manipulating existing medications and driving the optimization of experimental compounds. Moreover, considering the high attrition of drug candidates, application of PAL in early-stage drug discovery efforts could represent a powerful tool to detect, manage, and solve compounds’ liabilities and, in turn, yield better suited and safer therapeutic options.

In conclusion, PAL could represent a powerful strategy to improve our current knowledge of parasitic diseases and optimize the drug discovery and development process. Indeed, by leveraging the recent advances in the field and integrating old and new information, this approach has the potential to further feed the recent impetus in this area, facilitating more focused discovery and the development of effective and safe antiprotozoal drugs.

## Data Availability

No new data were created or analyzed in this study. Data sharing is not applicable to this article.

## Supplementary Materials

The following supporting information can be downloaded at: https://www.mdpi.com/article/10.3390/ph18010028/s1, Table S1: Antiprotozoal activity (*Trypanosoma brucei*, *Leishmania*, and *Trypanosoma cruzi*) and cytotoxicity of compounds and probes reported in Section 4 of the present review; Table S2: IC_50_ values of compounds reported in Section 6 of the present review displaying antimalarial activities.
