# Supplementary material for: Uncovering the Mechanism of Action of Antiprotozoal Agents: A Survey on Photoaffinity Labeling Strategy
Source: Pharmaceuticals (Basel). 2024 Dec 28;18(1):28. doi: 10.3390/ph18010028 (PMC11768348; doi:10.3390/ph18010028)
Supplement: Supplementary file 1 [file pharmaceuticals-18-00028-s001.zip › pharmaceuticals-3372499-supplementary.pdf]

Supplementary Materials

Uncovering the mechanism of action of antiprotozoal agents: a survey on photoaffinity labeling strategy.

Alessandro Giraudo[1,*], Cristiano Bolchi[1], Marco Pallavicini[1], Roberto Di Santo[2], Roberta Costi[2] and Francesco Saccoliti[3,*]

[1] Dipartimento di Scienze Farmaceutiche, Università degli Studi di Milano, via Mangiagalli 25, I-20133 Milano, Italy

[2] Istituto Pasteur-Fondazione Cenci Bolognetti, Dipartimento di Chimica e Tecnologie del Farmaco, "Sapienza" Università di Roma, p.le Aldo Moro 5, I- 00185 Rome, Italy

[3] Dipartimento di Scienze della Vita, della Salute e delle Professioni Sanitarie, Università degli Studi "Link Campus University", Via del Casale di S. Pio V 44, I- 00165 Rome, Italy

* Correspondence: Francesco Saccoliti, f.saccoliti@unilink.it; Alessandro Giraudo, alessandro.giraudo@unimi.it

Table S1. Antiprotozoal activity (*Trypanosoma brucei*, *Leishmania*, and *Trypanosoma cruzi*) and cytotoxicity of compounds and probes reported in Section 4 of the present review.

Compound/Probe	Molecular Weight	EC50 (µM)				
		Tb [1]	*L* [2]	*Tc* [3]	CC50 (µM) [4]	Reference
**Chamuvarinin**	604.91	1.4 (BSF)<br>1.9 (PF)	-	-	2.0 (HeLa)	[152]
**5**	407.60	1.8 (BSF)<br>8.4 (PF)	-	-	7.0 (HeLa)<br>7.0 (Vero)	[152]
**6**	562.72	13.0 (BSF)[5]<br>17.5 (BSF, *Tbb*)[6]<br>16.1 (PF)[5]<br>25.7 (PF, *Tbb)*[6]	20.3 (PRO, *Lm*)	51.2 (EP)	21.3 (HeLa)<br>21.3 (Vero)	[152]
**7**	511.67	11.8 (BSF)[5]<br>10.1 (BSF, *Tbb*)[6]<br>8.9 (PF)[5]<br>8.5 (PF, *Tbb*)[6]	32.6 (PRO, *Lm*)	29.9 (EP)	20.9 (HeLa)	[152], [166]
**8**	466.96	0.0389 (BFS, *Tbb*)	-	-	0.0108 (HEK293)	[164]
**9**	488.93	0.2653 (BSF, *Tbb*)	-	-	0.0646 (HEK293)	[164]
**10**	591.79	4.6 (BSF, *Tbb*)<br>3.5 (PF, *Tbb*)	15.6 (PRO, *Lm*)<br>17.7 (IA, *Ld*)	29.8 (EP)	12.1 (HeLa)<br>16.5 (Vero)<br>23.9 (THP-1)	[166]
**11**	472.59	13.0 (BSF, *Tbb*)<br>16.1 (PF, *Tbb*)	31.5 (PRO, *Lm*)	59.6 (EP)	-	[166]
**12**	511.67	11.8 (BSF, *Tbb*)<br>8.9 (PF, *Tbb*)	25.1 (PRO, *Lm*)	44.2 (EP)	-	[166]
**17-AAG**	585.70	-	IC_{50} =0.211 (PRO, *Lmx*)	-	-	[174]
**17-mADAG**	665.79	-	IC_{50} = 0.640 (PRO, *Lmx*)	-	-	[174]
**Actinoallolide A**	564.76	0.0083 (*Tbb*)<br>0.152 (*Tbr*)	-	0.400	-	[175], [176]

**13**	823.76	-	-	-	-	[175], [176]

[1] Activity profile on bloodstream (BSF) and procyclic (PF) forms of *T. brucei brucei* (*Tbb*) and *T. brucei rhodesiense* (*Tbr*) parasites expressed as EC_{50} (or IC_{50}, when specified). [2] Activity profile on promastigote (PRO) and intramacrophage amastigote (IA) forms of *L. major* (*Lm*), *L. donovani* (*Ld*) and *L. mexicana* (*Lmx*) parasites expressed as EC_{50} (or IC_{50}, when specified). [3] Activity profile on epimastigote (EP) form of *T. cruzi* parasite expressed as EC_{50} (or IC_{50}, when specified). [4] Cytotoxicity assessed on mammalian cells indicated in brackets and expressed as CC_{50}. [5] Activity reported in ref. [152]. [6]Activity reported in ref. [166].

Table S2. IC_{50} values of compounds reported in Section 6 of the present review displaying antimalarial activities.

Compounds	Molecular Weight	*P. falciparum* strain	IC_{50} (nM)	Reference
Artesunate (ATS)	384.42	3D7	5.96	[197]
APP	418.49	3D7	0.64	[197]
Chloroquine	319.88	3D7	34<br>19.48	[200]<br>[202]
		FAC8	156	[200]
ASA-Q	461.57	3D7	39	[200]
		FAC8	73	[200]
CQP	623.20	3D7	184.10	[202]
Mefloquine	378.32	K1<br>K1 Mef<br>D10	349<br>1083<br>826	[208]
ASA-MQ	544.58	K1<br>K1 Mef<br>D10	53<br>185<br>159	[208]
Plasmodione	330.31	Dd2	20	[209]
		NF54	46.6	[210]
**18**	286.33	Dd2	49	[209]
**19**	300.31	Dd2	1806	[209]
**20**	375.34	Dd2	417	[209]
**22**	304.32	NF54	179.2	[210]
ACT-186128	737.87	3D7	14.9	[214]
ACT460953	1384.63	3D7	34.1	[214]
Guttiferone A	602.80	3D7	5100	[217]
AZC-GA	844.00	3D7	5400	[217]
DDD01035881	404.30	NF54 gametocytes	292	[219]
**23**	384.41	NF54 gametocytes	3981	[219]
**24**	477.17	NF54 gametocytes	785	[219]
**25**	514.66	D10	0.77	[221]
		W2	0.91	[221]
**26**	646.70	D10	1021.51	[221]
		W2	1245.03	[221]
**27**	524.65	D10	207.78	[221]
		W2	195.96	[221]
**28**	516.63	D10	270.96	[221]
		W2	299.58	[221]
**29**	516.63	D10	15.03	[221]
		W2	19.48	[221]
**30**	516.63	D10	165.69	[221]
		W2	204.33	[221]
BIX01294	490.64	3D7	43.4<br>50	[222]<br>[224]
**31**	520.67	3D7	42	[224]
Dioncophylline A	377.48	FCBR	381	[228]
**32**	919.22	FCBR	45	[228]

**33**	738.98	FCBR	602	[228]
**34**	1070.25	FCBR	960	[228]
Albitiazolium	469.77	3D7	4.2	[232]
UA1936	640.90	3D7	4.5	[232]
